# Morphometric and genetic characterization as tools for selection of *Apis mellifera* (Hymenoptera: Apidae) stocks in an area of natural hybridization in Argentina

**DOI:** 10.3389/finsc.2022.1073999

**Published:** 2023-01-17

**Authors:** Leonardo Litvinoff, Francisco Menescardi, Leonardo Porrini, Romina Russo, María Clara Liendo, Alejandro Nucci, Esteban Lusarreta, Rocio Ventura, Luna Espasadin, A. Carolina Monmany-Garzia, Alejandra C. Scannapieco, Alberto Galindo-Cardona

**Affiliations:** ^1^ Reinas del Litoral SRL, San Salvador, Entre Ríos, Argentina; ^2^ Centro de investigación en abejas sociales, Instituto de Investigaciones en Producción, Sanidad y Ambiente (IIPROSAM-CONICET), Universidad Nacional de Mar del Plata, Mar del Plata, Argentina; ^3^ Instituto de Geneítica “E. A. Favret”, Instituto Nacional de Tecnologiía Agropecuaria (INTA)-Grupo vinculado al Instituto de Agrobiotecnologiía y Biología Molecular (IABIMO-CONICET), Buenos Aires, Argentina; ^4^ Consejo Nacional de Investigaciones Científicas y Técnicas, Tucumán, Argentina; ^5^ Instituto de Entomología, Fundación Miguel Lillo, Tucumán, Argentina; ^6^ Instituto de Ecología Regional (Universidad Nacional de Tucumán-CONICET), Yerba Buena, Argentina

**Keywords:** hybrid zone, mating, drone congregation areas, honey bees, Africanization

## Abstract

Beekeepers around the world select bees’ characteristics that facilitate and favor production. In regions where hybridization among lineages is taking place, this selection is a challenge, given that these regions are “natural laboratories”, where the action of evolutionary processes of a population or species occurs in real time. A natural honeybee (*Apis mellifera*) hybrid zone exists in Argentina between 28° and 35° South, where Africanized (AHB) and European (EHB) populations converge. In this zone, beekeepers use selected genetic resources of European origin mostly, since the local Africanized bees show a higher defensive behavior, which is not desirable for management. Although EHB colonies have many advantages for honey production, they are not fully adapted to the subtropical climate and are susceptible to certain parasitosis such as varroosis. In addition, both AHB and EHB mate in drone congregation areas (DCAs), where males and virgin queens fly to meet, resulting in variability in the desired characteristics. In this study, we explored the degree of hybridization within a DCA and its reference apiary, located in the province of Entre Ríos, by applying two complementary techniques. First, morphotypes with different degrees of hybridization between European and African subspecies were observed in the reference apiary, indicating a high sensitivity of this morphometric approach to detect hybridization in these populations. Second, a genetic analysis revealed haplotypes of both origins for drones in DCAs, with a higher prevalence of European haplotypes, while all the colonies from the reference apiary exhibited European haplotypes. Overall, our results are in line with the strong impact that commercial beekeeping has on the genetics of DCAs. We show how wing morphometry may be used to monitor hybridization between European and African subspecies, a tool that may be evaluated in other regions of the world where hybridization occurs.

## Introduction

Globally, there are geographical zones in which individuals of different genetic composition, distinguishable by one or several hereditary traits, meet and interbreed to produce hybrid offspring (1). These hybridization zones are considered “natural laboratories”, where it is possible to analyze the action of evolutionary processes of a population or species in real time (2). In addition, they are reservoirs of genotypic and phenotypic variability that are key for the evolution of certain traits that may eventually contribute to speciation processes (3). An interesting example of hybridization in real time is the process of Africanization that occurred in *Apis mellifera* populations in the American continent during the 20th century. European lineages of *A. mellifera* had been introduced for the first time in America in the beginning of the 18th century (4). The subsequent accidental introduction and release of the African subspecies *A. m. scutellata* in Brazil resulted in hybridization events that gave rise to Africanized populations, which rapidly spread throughout the continent (5). Indeed, in 50 years, the Africanized bees expanded throughout the Americas, from northern Argentina to the southern United States (6).

The mating process in *A. mellifera*, which is key in hybridization, occurs in drone congregation areas (DCAs) (7), where drones gather to mate with virgin queens. Since this species is polyandrous, a queen can mate with up to approximately 70 drones in the different fertilization flights (8). These natural areas are present in those places where honey bees exist and, in many cases, bees of different subspecies are found in them (9). Drones are attracted to DCAs mainly following the queen’s sex pheromone (7) or other drones´ pheromones (10, 11). In field observations, it has been detected that the buzz perceived at DCAs can also be an important attractive signal for other drones (12). Bees mate in flight, between 15 and 60 meters above the ground, flying at 12 kilometers per hour (13–15) and they are faithful to these sites, visiting them each mating season (9, 12, 16), although the mechanism that promotes such persistence is unknown (12, 14, 17). Virgin queens can travel approximately between three to 15 km away from their colonies to encounter hundreds of drones in DCAs (8, 18) and visit multiple DCAs close to each other and to their colony (19). Studies of African populations of *A. m. scutellata* have shown that DCAs are dynamic systems that concentrate a high genetic diversity and that present temporary genetic differentiation and a variable effective population size, probably due to a high turnover of wild colonies in the vicinity (20). In Mexico, through molecular analysis, it was found that there is seasonal variation in the presence of AHB and EHB drones at the DCA, with EHB drones coming out to the DCA at the end of the season and the AHB drones, at the beginning (21). At the same time, Collet et al. (22) evaluated the genetic structure of DCAs and commercial apiaries of Africanized and European origin in southern Brazil. Using microsatellite markers, they found a high genetic similarity between the colonies at the commercial apiary and the DCAs near them, and differences in the genetic structure of DCAs close to Africanized vs. European apiaries. Similar results were found by Mortensen & Ellis (23) in DCAs in the United States. In Argentina, at least 20 DCAs have been genetically and environmentally characterized in different ecoregions (12, 24).

Different races of *A. mellifera* that may encounter at hybridization regions show variability in wing morphometry, a character that varies genotypically and phenotypically and thus represents a useful tool for subspecies´ classification. The use of morphometric techniques, that allow obtaining automated measurements of the patterns of variation in venous branches of the *A. mellifera* forewing, has been widely used both to distinguish African from European subspecies and to characterize the evolutionary lineages of *A. mellifera* with a high degree of consistency (25–28). The analysis of populations that are present in areas of natural hybridization, either through wing morphometry and/or genetics, provides valuable information for the characterization of this process. For instance, examining bee wing morphometry, Porrini et al. (29) added evidence of the existence of a latitudinal limit for Africanization in populations of *A. mellifera* in Buenos Aires, as previously suggested by Abrahamovich et al. (30).

Pioneer studies in the beekeeping region of Argentina revealed the existence of six lineages, both European and Africanized, in local populations, with the most frequent haplotype being C1 (*A. m. ligustica*) (30–32). Recently, Agra et al. (33) and Calfee et al. (34) confirmed the presence of north-south latitudinal clines between 32° and 39° North and 28° and 37° South for the level of hybridization between European and Africanized bee populations. They identified four groups (genetic clusters) that were explained not only by geographic distribution and degree of Africanization, but also by human influence through beekeeping activities. All the mentioned studies were based on the use of both mitochondrial and nuclear molecular markers as key tools for the identification of mitochondrial lineages and the characterization of genetic diversity.

In order to explore the degree of hybridization of bees at a DCA and in a queen mating yard as a reference apiary, we used two complementary techniques: characterization of subspecies (geometric morphometry of the wing) and characterization of the maternal lineage (mitochondrial DNA) of bees in Entre Ríos, Argentina. This approach helped us to describe local genetic resources in a comprehensive way, since it included the contribution of males to the process. In addition, it was useful to study the genetic variation in populations present at the DCA and colonies, which provide valuable information about the current status of Africanization in the region and is, therefore, key for the adjustment of breeding programs in support of a sustainable beekeeping development.

## Materials and methods

### Study area

**Figure 1:**
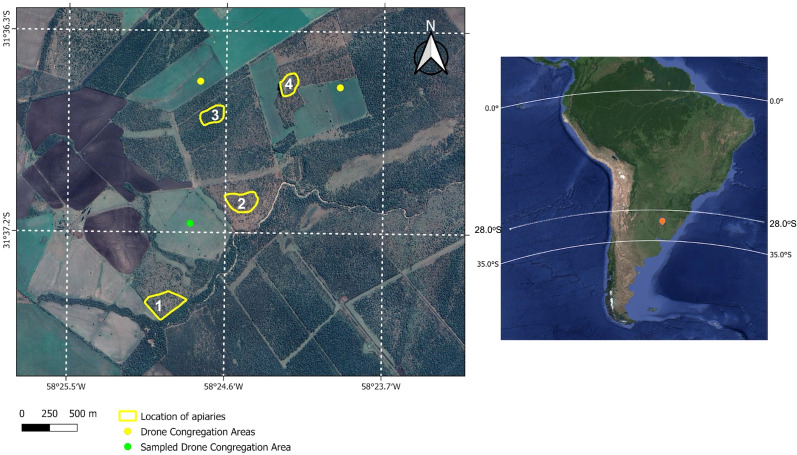
Updated Google satellite images of the study area (left: Map data: ©2019 Google) and South America (right: Map data: ©2019 Google, TerraMetrics). In the image of South America, the study area is marked with an orange marker; the hybridization zone occurs between 28° S and 35° S in Argentina. The image of the study area shows the three Drone Congregation Areas (DCAs) and the four apiaries included in this study and located near the city of San Salvador, Entre Ríos. The DCA from which the samples for this study were collected is marked in green. The samples for wing morphometry analyses were taken from apiaries 1, 2, and 3. Apiarieses 1 to 4 were used to observe the drones´ exit routes to the DCAs.

This study was performed in San Salvador, province of Entre Ríos, Argentina (31° 37’ 0’’ South, 58° 30’ 27’’ West, **Figure 1**). Entre Ríos occupies a large extension of the eastern boundary of the Pampas plain, with smooth slopes that rise to the west and north. This region of Argentina is characterized by bushy vegetation, with a humid temperate climate, and moderate conditions, which are suitable for the development of beekeeping activities (35). The average rainfall regime is approximately 1400 millimeters per year. The flora with interest for beekeepers is diverse, with woody shrubby and sub-shrub species, among which *chilcas (Baccharis salicifolia), chañares* (*Geoffroea* sp.), *molle (Bumelia obtusifolia), algarrobos* (*Prosopis* sp.), *aromitos* (*Acacia caven*), *tala* (*Celtis tala*), *ñandubay* (*Prosopis affinis*), and *coronillo* (*Scutia buxifolia*), are among the characteristic flora. The presence of species of beekeeping interest was critical, because they provided high-quality protein pollen and highly varied nectar composition during seven months (i.e., September to April). During this period, the flowering of red eucalyptus (*Eucalyptus saligna*) occurs first and the flowering of *chilcas* (*Baccharis salicifolia*) takes place in a second and more important flowering peak that ends in April (35). In this region main crops correspond to: corn, soybean, rice, and wheat, which are produced under intensive agricultural management. In addition, artificial meadows for livestock feeding are composed of species such as *alfalfa* (*Medicago sativa*), *white clover* (*Trifolium alba*), and *lotus* (*Lotus* sp.). Many “weeds” in these meadows constitute another food resource for honey bees and beekeeping, such as the thistle species: *Carduus acanthoides*, *cundidor* (*Cirsium arvense*), thistle (*Cynara cardunculus*), *asnal* (*Silybum marianum*) and *cardillo* (*Scolymus hispanicus*) (35).

The search and subsequent sampling of the DCAs was carried out in the queen bee ¨mating yard¨ of the Reinas del Litoral beekeeping facility (SENASA E-02). The mating yard included four apiaries, with a minimum distance of 1 km among them (see **Figure 1**). In two of the apiaries 900 mating nuclei and 200-220 support colonies were located, organized by the age of the queens. The mating yard was located on a smooth terrain, with absolute heights of 80 meters above sea level. The site was located in the limits of Chaco vegetation and humid Pampas; and included an 800- to 900- hectares forest fragment with naturalized and native species.

### Location of drone congregation areas

In order to locate the DCAs, we used a method based on the direct tracking of the entry and exit route of drones from the reference apiary. Apiaries 1 to 4 were taken as references (**Figure 1**). In October 2019, we made observations at the entrance of four colonies of apiary 2 and detected the starting orientation of the drones. We then walked around apiary 2 raising a helium-inflated latex balloon baited with a synthetic pheromone (9-hydroxy-2-enoic acid). Every time the bait was touched by one or more drones, we marked the geographic coordinates on a GPS (Global Positioning System) (12, 24, 36). This procedure was performed three times a day for three consecutive days and at three different points of the potential DCA to confirm the data and to determine its size. In addition, once the DCA was confirmed, we used an Unmanned Aerial Vehicle (UAV), to which we attached a pheromone bait, raising it repeatedly to capture the drones (see green dot in **Figure 1**). The capture of the drones was carried out using entomological nets in the DCA. The samples were kept in 96% alcohol and 20 drones were randomly taken for posterior genetic analysis. In the laboratory, the geographic coordinates taken with the GPS were used to map the DCA on updated satellite images (**Figure 1**).

### Wing geometric morphometry

For morphometric analyses, we sampled honey bees from 10 colonies located in apiaries 1, 2, and 3 (**Figure 1**). The first sampling was carried out in the fall of 2020 in five colonies: P 18, P 19, V 16, V 151 and V 152. The second sampling was carried out in spring 2020 from five colonies, of which two had been previously sampled: P19, V16, V16A, V16B, V1. The genetic lines (i.e., queen bees) that we analyzed were selected after two years of evaluation and continuous monitoring throughout the season, which occurred from September to the end of March. From each colony, 10 individuals were randomly selected and their left forewings were cut.

Wings were mounted on glass frames and scanned with a Plustek Opticfilm 8100 (LaserSoft Imaging, Kiel, Germany) (7200 dpi). Using the images obtained for each colony and those corresponding to each pure subspecies (*A. m. carnica, A. m. caucasica, A. m. iberiensis, A. m. intermissa, A. m. ligustica, A. m. mellifera* and *A. m. scutellata*, obtained from the Morphometric Bee Data Bank in Oberursel, Germany), 19 homologous points (26) were manually marked in tpsDIG v2.16 (37) and then clustered in tpsUtil v1.4 (38).

In the free software MORPHOJ v.1.07a (39), the alignment was performed using the Procrustes adjustment to minimize the differences due to size, position and rotation between all the landmark configurations (40). Canonical Variable Analysis (CVA) and Mahalanobis distances were calculated (41) to establish the degree of similarity between the analyzed colonies and the reference groups for each *A. mellifera* subspecies.

### Genetic analysis

For the identification of the mitochondrial lineage (i.e., European or Africanized) of drones (sampled at the DCA) and workers (collected from the interior of each colony), the PCR-RFLP technique was applied to the COI-COII gene region. This technique consists in the amplification of the gene region and subsequent digestion of the fragment with restriction enzymes. The protocol and primers described by Hall & Smith (42) and Lobo-Segura (43), with standardized modifications in the Laboratory of Insects of Agronomic Importance (Igeaf, INTA Castelar) were used. DNA extraction was performed individually from the thorax of drones and workers collected in the DCA or from the colonies, respectively. The quality and purity of the extracted DNA were confirmed by electrophoretic runs and measurement of reduced volumes in the spectrophotometer. The PCR amplification reaction was carried out using specific primers and conditions established according to Hall & Smith (42) and Lobo-Segura (43). The digestion of the amplified fragments was carried out by incubation with Hinf I enzyme (Promise, Madison, MN, USA). The restriction fragments separated into 3% agarose gels (weight/volume), were dyed with GelRed and photographed under UV light. The restriction patterns obtained were analyzed to assign the mitochondrial haplotype of each sample, considering the reference patterns established in the bibliography (33). The total number of analyzed drones from the DCA was 20. The total number of analyzed workers was 10, one per colony. The sampling of worker bees of the 10 colonies for genetic analysis and for morphometric analysis was carried out simultaneously.

## Results

### Location of drone congregation areas

After walking approximately 6 km and taking the apiaries of the ‘mating yard’ (24) as a reference, we found three DCAs (**Figure 1**). The maturation state of the drones sampled at the DCAs was determined, detecting 99% of the drones in a state of sexual maturity. This value was higher than those found for drones at DCAs in Tucumán (95%) and Buenos Aires (90%) for the same dates (unpublished data). In addition, we carried out maturity evaluations of drones in orphan colonies (i.e., colonies producing queen cells) located 8 km from the DCAs, and found that 20% of those drones were immature, both in the periphery and in the center of the colony.

### Wing geometric morphometry

**Figure 2:**
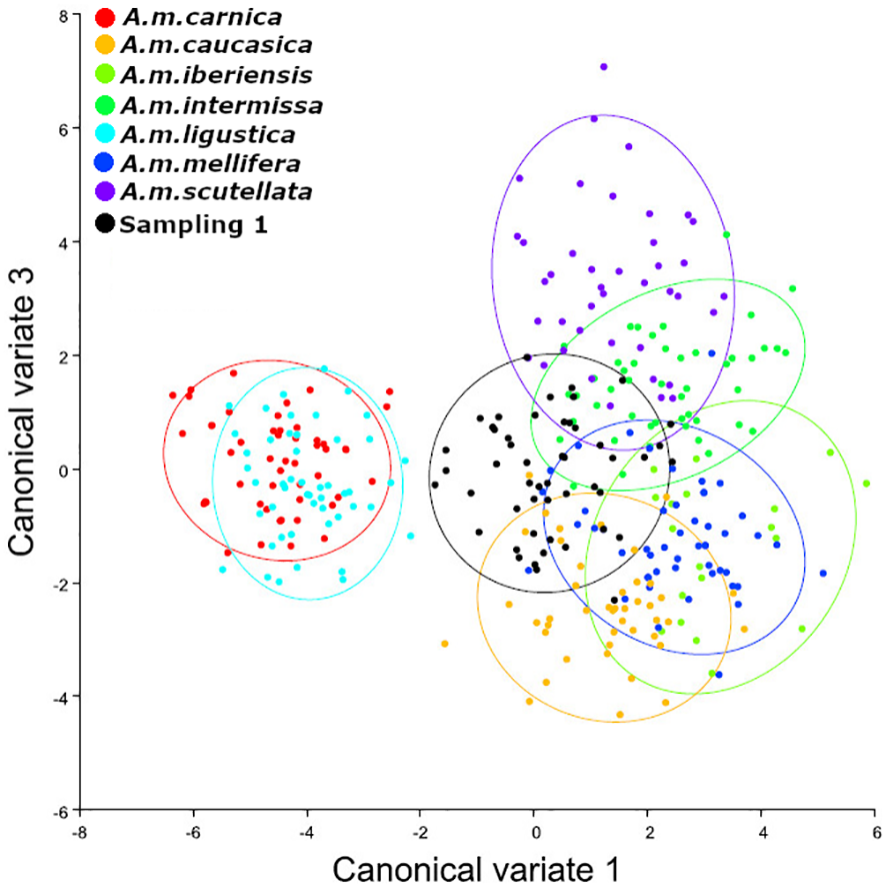
Analysis of Canonical Variables. Sampling 1 - Comparison to reference samples of *A. mellifera* subespecies (*A. m. carnica* = red; *A. m. caucasica* = orange; *A. m. iberiensis* = light green; *A. m. intermissa*= green; *A. m. ligustica* = turquese; *A. m. mellifera* = blue and *A. m. scutellata* = purple). The analyzed sample is represented in black (Reinas del Litoral). We used 10 individuals (wings) per colony and 50 individuals (wings) per reference group for each subspecies, except for *A. m. iberiensis*, which were 20.

**Figure 3:**
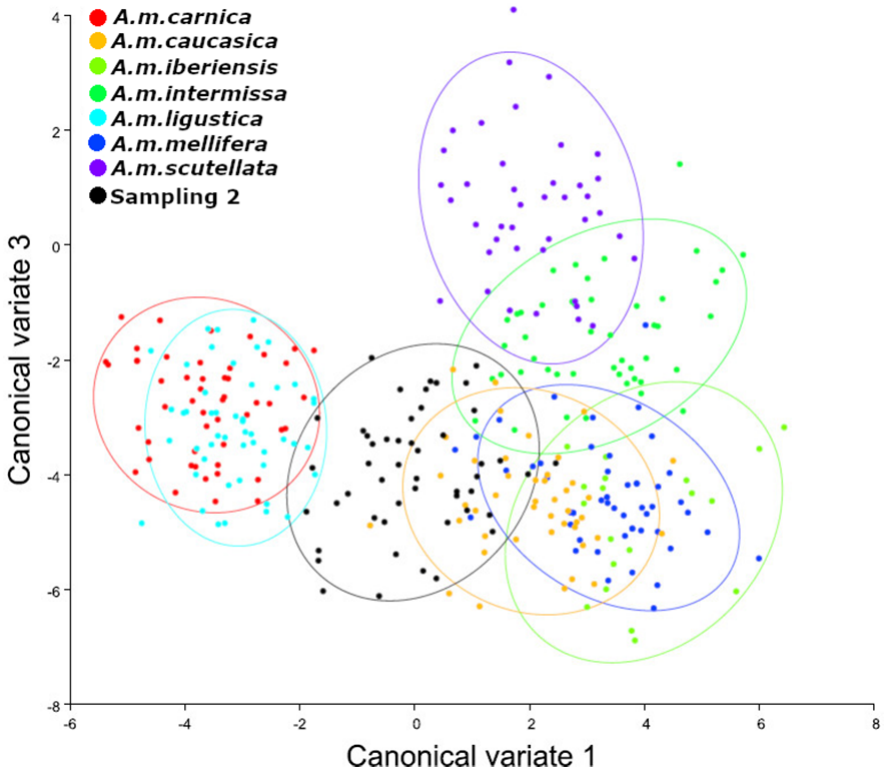
Analysis of canonical variables. Sampling 2 - Comparison to reference samples of *A. mellifera* subespecies (*A. m. carnica* = red; *A. m. caucasica* = orange; *A. m. iberiensis* = light green; *A. m. intermissa*= green; *A. m. ligustica* = turquese; *A. m. mellifera* = blue and *A. m. scutellata* = purple). The analyzed sample is represented in black (Reinas del Litoral). We used 10 individuals (wings) per colony and 50 individuals (wings) per reference group for each subspecies except for *A. m. iberiensis*, which were 20.

We observed that the bees sampled in autumn presented an Africanized morphotype, with different degrees of hybridization between subspecies and greater similarity (lower Mahalanobis distances) to the subspecies *A. m. caucasica, A. m. intermissa* and *A. m. scutellata* (**Table 1**, **Figure 2**). The second sampling (spring) indicated a greater degree of hybridization among subspecies (**Table 2**, **Figure 3**), compared to the autumn sampling and greater similarity to European subspecies belonging to the C lineage (*A. m. carnica*, *A. m. caucasica*, *A. m. ligustica*).

**Table 1:** Mahalanobis distances.

Bees subespecie	Sample 1 - 07/03/2020
*A. m. carnica*	5,945
*A. m. caucasica*	4,809*
*A. m. iberiensis*	6,244
*A. m. intermissa*	4,679*
*A. m. ligustica*	5,772
*A. m. mellifera*	5,419
*A. m. scutellata*	5,027*

Sampling 1- Genetic distances of the samples to each pure subspecies of Apis mellifera (*A. m. carnica, A. m. caucasica, A. m. iberiensis, A. m. intermissa, A. m. ligustica, A. m. mellifera, and A. m. scutellata*). * indicates the smallest genetic distances.

**Table 2:** Mahalanobis distances.

Bees subespecie	Sample 2 - 15/12/2020
*A. m. carnica*	5,256*
*A. m. caucasica*	5,320*
*A. m. iberiensis*	6,425
*A. m. intermissa*	5,451*
*A. m. ligustica*	5,450*
*A. m. mellifera*	5,850
*A. m. scutellata*	5,973

Sampling 2 - Genetic distances of the samples to each pure subspecies of Apis mellifera (*A. m. carnica, A. m. caucasica, A. m. iberiensis, A. m. intermissa, A. m. ligustica, A. m. mellifera, and A. m. scutellata*). * indicates the smallest genetic distances.

If we analyze the results of morphometric analysis within each colony (**Supplementary Table 1** and **Figure 1**) we can observe that although most colonies presented greater similarity to the subspecies *A. m. caucasica* and *A. m. intermissa*, there was variability in relation to the rest of the subspecies included in the analysis. In autumn, three colonies (P18, V151, V152) showed smaller Mahalanobis distances to *A. m. scutellata*, than to the other subspecies, revealing a predominantly African morphotype. On the other hand, in spring three colonies (P19, V16 and V16B) showed smaller distances to *A. m. carnica* and *A. m. ligustica* than to the other subspecies and one (V16a) showed a small distance to *A. m. mellifera*, revealing a predominantly European morphotype. In both seasons (autumn and spring), a high degree of hybridization was observed and there was no dominance of one subspecies over the others (i.e., Mahalanobis distances were quite similar).

**Figure 4:**
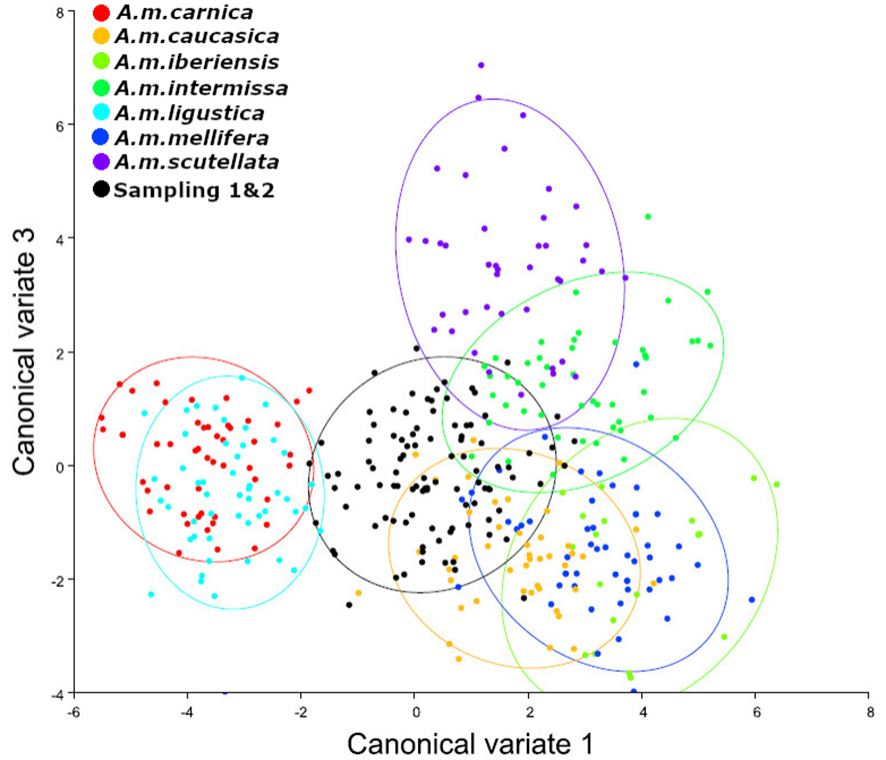
Canonical variate analysis (CVA). The first two canonical variates illustrate wing shape variation in Sampling 1 and 2 (black color), in comparison to reference samples of *A. mellifera* subespecies (*A. m. carnica* = red; *A. m. caucasica* = orange; *A. m. iberiensis* = light green; *A. m. intermissa*= green; *A. m. ligustica* = turquese; *A. m. mellifera* = blue and *A. m. scutellata* = purple). Each marker (points) represents the mean scores of each colony. The ellipses represent 95% confidence intervals around the centroid of each data cluster. We used 10 individuals (wings) per colony and 50 individuals (wings) per reference group for each subspecies except for *A. m. iberiensis*, which were 20.

When both seasons were analyzed together, it was observed that *A. m. intermissa, A. m. caucasica*, and *A. m. scutellata* were the three subspecies with the lowest Mahalanobis distances (**Table 3**, **Figure 4**). However, distances to the rest of the subspecies were not much higher, and we cannot affirm that the predominant morphotype in the total samples was only African.

**Table 3:** Mahalanobis distances.

Bees subespecie	Samples 1 and 2 - 07/03/2020 y 15/12/2020
*A. m. carnica*	5,187
*A. m. caucasica*	4,757*
*A. m. iberiensis*	6,159
*A. m. intermissa*	4,683*
*A. m. ligustica*	5,231
*A. m. mellifera*	5,365
*A. m. scutellata*	5,072*

Sampling 1 and 2 - Genetic distances of the samples to each pure subspecies of Apis mellifera (*A. m. carnica, A. m. caucasica, A. m. iberiensis, A. m. intermissa, A. m. ligustica, A. m. mellifera, and A. m. scutellata*). * indicates the smallest genetic distances.

### Genetic analysis

**Figure 5:**
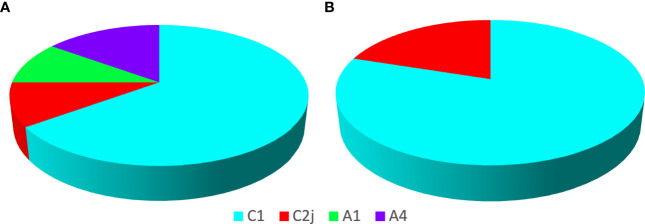
Genetics in the drone congregation area and in the reference apiary. Percentages of mitochondrial haplotypes detected in the *Apis mellifera* drones sampled at the DCA **(A)** and in the workers of the 10 colonies at the reference apiary **(B)**. C1 and C2j correspond to European haplotypes, while A1 and A4 correspond to haplotypes of African origin. Twenty individuals (drones) per sampled DCA and one individual (worker) per reference hive were used.

Haplotypes of European and African origin were found at the DCA (**Figure 5A**). Seventy-five percent of the analyzed drones showed a European mitochondrial origin (Haplotype C), being C1 (65%) more frequent and C2J (10%), the least frequent (**Figure 5A**). Haplotype C corresponds to the evolutionary lineage of Eastern Europe, where the Italian honey bees *A. m. ligustica* and the carniolan bee, *A. m. carnica*, are included. Twenty-five percent of the remaining drones showed an African mitochondrial origin (Haplotype A), with similar frequencies for A1 (10%) and A4 (15%) (**Figure 5A**). These haplotypes are generally associated with the subspecies *A. m. intermissa* and *A. m. scutellata*, respectively. Regarding the results of the reference apiary, 100% of the colonies showed a European mitochondrial origin, with C1 being the most prevalent (80%) and C2J (20%), the least prevalent in both samples (**Figure 5B**).

## Discussion

In this study we identified three new DCAs for Argentina. The characterization of subspecies using wing geometric morphometry showed a variable bee morphotype, which included both European and African subspecies, and different degrees of hybridization. In this sense, the morphometric approach allowed us to reveal a considerable representation of hybrids in the colonies and its implementation is necessary to describe whether the pattern in drones at the DCA is similar. The characterization of the maternal lineage (mitochondrial DNA) in drones collected in one DCA evidenced the presence of haplotypes of both African and European origin, with the highest prevalence of the latter. Congruently, we detected the exclusive presence of European lineages (C1 and C2J) in the bees at the reference apiary.

### Location of drone congregation areas

The DCAs located in this study presented landscape characteristics that were similar from other DCAs in Argentina, such as simple vegetation structure (i.e., grasslands and open areas) and undulated terrain with low slope.

The high percent of mature drones found in the DCA could be due to the fact that the sampling was carried out at the beginning of the mating season, when the population is reduced due to the lack of floral resources. Alternatively, climatic conditions prior to the mating season may hasten the development of drone-producing colonies (personal observation, LL and FM). In the colonies producing queen cells, we also found a high percentage of drone maturity. Beekeepers breeding queens have observed massive entries of drones in this type of colony. A plausible explanation of this pattern is that drones emit pheromones that attract other mature drones (11, 44) from nearby colonies or they may come from orphan colonies and freely enter to feed (45). In addition, virgin queens emit “quacks” sounds after emergence to warn the workers that they are about to leave the colony and to inhibit the exit of the other queens (46). This sound could generate drone clumping around cells of queens that are close to emergence (personal observation, LL and FM). We observed that queen cells were surrounded by several drones and that these colonies exhibited a high number and phenotypic diversity of mostly mature drones. This behavior suggests that the presence of the drones could be a stimulus for the development of queens that are still within their cells, a hypothesis that must be tested experimentally. We conclude that the beginning of the mating season is an appropriate temporal window to manage queens’ reproduction at the DCAs selecting drones with desired characteristics.

### Wing geometric morphometry

The variability found in the morphotypes that we analyzed is consistent with previously obtained results in managed populations in Argentina (29, 47). In transition areas between European and Africanized populations, where gene flow is extensive, differences at the mitochondrial, nuclear, and morphometric levels are generally observed because they represent hybrid zones (31, 32). Colonies in these transition zones often exhibit African features, even in areas where relatively high proportions of European alleles persist. The dominance of African alleles does not necessarily result in the loss of European markers, but contributes to the preservation of the African phenotype (Scott 48). On the other hand, although the high intra-colonial diversity that we found could be conferring to the colonies a higher degree of resistance to certain pathogens and/or greater productivity in adaptive terms (49–51), we believe that it is necessary to evaluate other factors in the future, such as population management, the type of multiplication used, and the flow of resources in different regions and times of the year, in order to advance in such a hypothesis.

The most interesting results when comparing wing shape are observed at the colony level. The high intra-colony variation here detected must be further evaluated in different regions and time scales to provide queen breeders a rapid genetic characterization of their stocks for selection and multiplication. As future perspectives, we believe it is important to explore the variability of drone morphotypes at DCAs. A limitation regarding this type of analysis is that, unlike worker bees, no image bank of drone wings is available as a reference for the different subspecies of *A. mellifera*. However, it is possible to compare the morphotype of drones in congregation areas with drones from nearby managed colonies (25).

### Genetic analysis

Our results indicated a high homogeneity in the mitochondrial origin of the colonies within the reference apiary, since all exhibited European mitochondrial haplotype. Our results are consistent with the fact that beekeeping in this area of ​​the province of Entre Ríos is carried out mainly with materials of European origin, since beekeepers maintain the use of this genetics through the implementation of management strategies such as queen and bees breeding programs (33). In the DCA we detected a genetic pattern that was similar to that observed in Buenos Aires, and contrasting with what was observed for the province of Tucumán, where the DCAs had a high representation of drones of African origin (12). Although the pattern found in Entre Ríos indicated a high proportion of drones of European origin, it was possible to detect 25% of African mitochondrial haplotypes (A1 and A4); these values are ​​significantly higher than those observed in the DCA in Buenos Aires (3%; 12). Studies about the proportion of African vs. European lineages within DCAs in other regions of the world are limited to that of Mortensen and Ellis (23) in a non-hybridization region of the USA, who showed that this proportion favored African lineages when distance to European managed colonies increased. Despite the impact that beekeeping can have on the dynamics and genetics of bees at the DCA, these sites maintain a higher haplotype diversity compared to their source reference apiaries, suggesting the importance of protecting these sites as reservoirs of genetic variability for populations of the species.

In Mexico, a partial seasonal isolation in the reproduction of African-derived and European drones in the vicinity of a DCA (21) was reported. Our findings detected that in both the DCA and the reference apiary, the EHB haplotypes predominated at the beginning of the season. A potential seasonal variation in haplotype frequency must be further evaluated in Argentina and compared to the pattern found in the north hemisphere.

Mortensen and Ellis (23) showed that the proportion of drones of a particular genetic origin at the DCAs can be affected by drones “flooding”. This technique could have a strong influence on selective breeding programs since it would allow to partially control the paternal contribution in the breeding populations of honey bees (23, 52). In this sense, the relatively low abundance of African haplotypes in the DCA in this study could be the product of the high influence of the reference apiary and other surrounding commercial apiaries, which multiply genes of European origin almost exclusively. Thus, it is key to design strategies that appropriately monitor the genetic diversity of bees at DCAs at different moments during the mating season, both for application toward decision-making and beekeeping management actions, and to answer eco-evolutionary questions such as understanding the mechanisms leading to the cooperation of bees´ populations of diverse origin (e.g. Africanized, European, local ecotypes) in the formation of a DCA.

The convergence of AHB and EHB at hybridization sites has been a topic of much interest in gene flow research in *Apis melifera*. More sophisticated methods than those used here, such as microsatellites or SNPs, showed better genetic resolution at these sites (33, 34). It is important to highlight, however,that by using less expensive molecular and morphometric techniques, similar results can be obtained (53–56). In conclusion, our results support the existence of hybrid populations based on a variable morphotype comprising both European and African subspecies, and on the mitochondrial lineage. Drone congregation areas represent interesting study areas that allow evaluating locally adapted bee populations and their natural evolution through time. These DCAs play a crucial role in the survival of bees, which are involved in pollination and are essential for our food security. In addition, we highlight the need to protect these mating areas globally, due to their value as genetic reservoirs (1) and to take advantage of them for the evaluation of living materials from the region.

## Data availability statement

The original contributions presented in the study are included in the article/**Supplementary Material**. Further inquiries can be directed to the corresponding authors.

## Author contributions

AG-C and AS: methodology and analysis, conceptualization, validation and visualization, resources and project administration, supervision and writing—original draft preparation. AG-C, AS, LL, FM, LP, RR, ML, AN, EL, RV, LE, and AM-G: investigation and writing—review and editing. LP and AN, EL, RV: software and data curation. All authors have read and agreed to the published version of the manuscript.
