# Supplementary figures and images for: Morphometric and genetic characterization as tools for selection of Apis mellifera (Hymenoptera: Apidae) stocks in an area of natural hybridization in Argentina
Source: Front Insect Sci. 2023 Jan 17;2:1073999. doi: 10.3389/finsc.2022.1073999 (PMC10926486; doi:10.3389/finsc.2022.1073999)

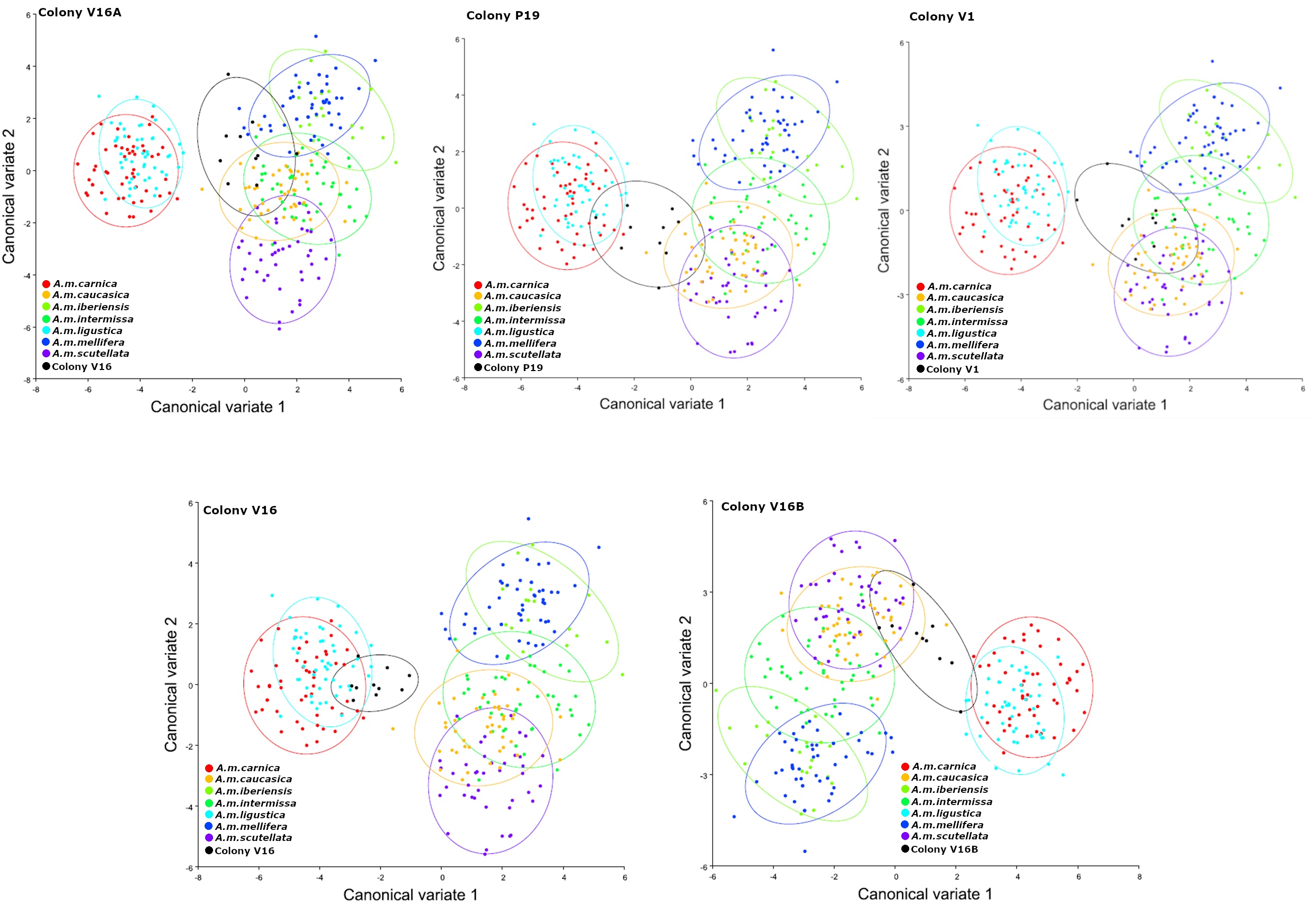

Supplement: Supplementary file 1 [file Image_1.jpg]
