# Supplementary material for: Morphometric and genetic characterization as tools for selection of Apis mellifera (Hymenoptera: Apidae) stocks in an area of natural hybridization in Argentina
Source: Front Insect Sci. 2023 Jan 17;2:1073999. doi: 10.3389/finsc.2022.1073999 (PMC10926486; doi:10.3389/finsc.2022.1073999)
Supplement: Supplementary file 2 [file Presentation_1.pdf]

Supp. Material 3: Manuscript translation to Spanish

Litvinoff et al. 2022. Morphometric and genetic characterization as tools for selection of *Apis mellifera* (Hymenoptera: Apidae) stocks in an area of natural hybridization in Argentina. ***Frontiers in Insect Science***. doi: 10.3389/finsc.2022.1073999

Autores responsables: Alberto Galindo-Cardona y Alejandra Scannapieco

Teléfono: +54 381 640 8826

Correo electrónico: agalindo@conicet.gov.ar; scannapieco.a@inta.gob.ar

Título: **Caracterización morfométrica y genética como herramientas para la selección de poblaciones de *Apis mellifera* (Hymenoptera: Apidae) en un área de hibridación natural en Argentina**

Autores: Leonardo Litvinoff[1]; Francisco Menescardi[1]; Leonardo Porrini[2]; Romina Russo[3]; Maria C. Liendo[3,5]; Alejandro Nucci[2]; Esteban Lusarreta[2]; Rocio Ventura[2]; Luna Espasadin[6]; A. Carolina Monmany-Garzia[4]; Alejandra C. Scannapieco[3,5] and Alberto Galindo-Cardona[5,6]

Filiaciones: [1]*Reinas del Litoral SRL.*

[2]*Centro de investigación en abejas sociales, Instituto de Investigaciones en Producción, Sanidad y Ambiente (IIPROSAM-CONICET), Universidad Nacional de Mar del Plata, Argentina.*

[3]*Instituto de Genética "E. A. Favret", Instituto Nacional de Tecnología Agropecuaria (INTA)-Grupo vinculado al Instituto de Agrobiotecnología y Biología Molecular (IABIMO-CONICET), Buenos Aires, Argentina.* [4]*Instituto de Ecología Regional (Universidad Nacional de Tucumán-CONICET), Yerba Buena, Argentina.*

[5]*Consejo Nacional de Investigaciones Científicas y Técnicas, Tucumán, Argentina.*

[6]*Fundación Miguel Lillo, Tucumán, Argentina*

Leonardo Litvinoff: leonardolitvinoff@gmail.com;

Francisco Menescardi: Cari7a@hotmail.com;

Leonardo Porrini: leoporrini@gmail.com; 0000-0002-2480-9635

Romina Russo: russo.romina@inta.gob.ar; 0000-0001-6888-9123

Maria C. Liendo: liendo.clara@inta.gob.ar; 0000-0002-8464-5652

Alejandro Nucci: nucciale44@gmail.com;

Esteban Lusarreta: estebanlusarreta.mdq@gmail.com; 0000-0001-5111-441X

Rocio Ventura: venturarociom@gmail.com; 0000-0003-2624-8365

Luna Espasadin: luniespasadin@gmail.com;

A. Carolina Monmany-Garzia: acmonmany@conicet.gov.ar; 0000-0003-0240-719X

Alejandra C. Scannapieco: scannapieco.a@inta.gob.ar; 0000-0002-4228-2996

Alberto Galindo-Cardona: agalindo@conicet.gov.ar, 0000-0003-3896-1934

Resumen. Los apicultores de todo el mundo seleccionan las características de las abejas que facilitan y favorecen la producción. En las regiones donde se produce hibridación entre linajes, esta selección constituye un desafío, dado que estas regiones son "laboratorios naturales", donde la acción de los procesos evolutivos de una población o especie ocurre en tiempo real. En Argentina existe una zona de hibridación natural de la abeja melífera (*Apis mellifera*) entre los 28º y 35º Sur, donde confluyen poblaciones africanizadas (AHB) y europeas (EHB). En esta zona, los apicultores utilizan mayoritariamente recursos genéticos seleccionados de origen europeo, ya que las abejas africanizadas locales muestran un mayor comportamiento defensivo, lo que no es deseable para su manejo. Aunque las colonias EHB presentan muchas ventajas para la producción de miel, no están totalmente adaptadas al clima subtropical y son susceptibles a ciertas parasitosis, como la varroasis. Además, tanto las AHB como las EHB se aparean en áreas de congregación de zánganos (ACZ), donde los machos y las reinas vírgenes vuelan para encontrarse, lo que da lugar a una variabilidad en las características deseadas. En este estudio, exploramos el grado de hibridación dentro de una ACZ y su apiario de referencia, ubicados en la provincia de Entre Ríos, mediante la aplicación de dos técnicas complementarias. En primer lugar, se observaron morfotipos con diferentes grados de hibridación entre las subespecies europea y africana en el apiario de referencia, lo que indica una alta sensibilidad de este enfoque morfométrico para detectar la hibridación en estas poblaciones. En segundo lugar, un análisis genético reveló haplotipos de ambos orígenes para los zánganos de las ACZ, con una mayor prevalencia de haplotipos europeos, mientras que todas las colonias del colmenar de referencia presentaban haplotipos europeos. En conjunto, nuestros resultados concuerdan con el fuerte impacto que la apicultura comercial tiene en la genética de las ACZ. Mostramos cómo la morfometría alar puede utilizarse para monitorear la hibridación entre subespecies europeas y africanas, una herramienta que puede evaluarse en otras regiones del mundo donde se produce hibridación.

frontiers

Palabras clave: zona híbrida, apareamiento, áreas de congregación de zánganos, abejas melíferas, africanización.

INTRODUCCIÓN

A escala mundial, existen zonas geográficas en las que individuos de diferente composición genética, distinguibles por uno o varios caracteres hereditarios, se encuentran y se entrecruzan para producir descendencia híbrida (Barton y Hewitt 1985). Estas zonas de hibridación se consideran "laboratorios naturales", donde es posible analizar la acción de los procesos evolutivos de una población o especie en tiempo real (Harrison 1990). Además, son reservorios de variabilidad genotípica y fenotípica clave para la evolución de ciertos caracteres que eventualmente pueden contribuir a procesos de especiación (Endler 1986). Un ejemplo interesante de hibridación en tiempo real es el proceso de africanización que se produjo en las poblaciones de *Apis mellifera* en el continente americano durante el siglo XX. Los linajes europeos de *A. mellifera* habían sido introducidos por primera vez en América a principios del siglo XVIII (Sheppard 1989). La posterior introducción y liberación accidental de la subespecie africana *A. m. scutellata* en Brasil dio lugar a eventos de hibridación que originaron poblaciones africanizadas, que se extendieron rápidamente por todo el continente (Winston 1987). De hecho, en 50 años, las abejas africanizadas se expandieron por todo América, desde el norte de Argentina hasta el sur de Estados Unidos (Galindo-Cardona et al. 2013).

El proceso de apareamiento en *A. mellifera*, clave en la hibridación, se produce en áreas de congregación de zánganos (ACZ) (Withrow & Tarpy, 2018), donde los zánganos se reúnen para aparearse con reinas vírgenes. Dado que esta especie es poliándrica, una reina puede aparearse con hasta aproximadamente 70 zánganos en los diferentes vuelos de fecundación (Brutscher et al. 2019). Estos espacios naturales están presentes en aquellos lugares donde existen abejas melíferas y, en muchos casos, en ellos se encuentran abejas de

frontiers

diferentes subespecies (Koeniger et al. 2005). Los zánganos son atraídos a las ACZ principalmente siguiendo la feromona sexual de la reina (Withrow & Tarpy, 2018) o las feromonas de otros zánganos (Bortolotti & Costa, 2014; Villar et al. 2018). En observaciones de campo, se ha detectado que el zumbido percibido en las ACZ también puede ser una importante señal de atracción para otros zánganos (Galindo-Cardona et al. 2020). Las abejas se aparean en vuelo, entre 15 y 60 metros sobre el suelo, volando a 12 kilómetros por hora (Oertel 1956; Zmarlicki & Morse, 1963; Loper et al. 1992) y son fieles a estos sitios, visitándolos cada temporada de apareamiento (Tribe 1982; Koeniger et al. 2005; Galindo-Cardona et al. 2020), aunque se desconoce el mecanismo que promueve tal persistencia (Zmarlicki & Morse, 1963; Strang, 1970; Galindo-Cardona et al. 2020). Las reinas vírgenes pueden viajar aproximadamente entre tres y 15 km lejos de sus colonias para encontrarse con cientos de zánganos en las ACZs (Oxley & Oldroyd 2010, Brutscher et al. 2019) y visitar múltiples ACZs cercanas entre sí y a su colonia (Bastin et al. 2017). Estudios de poblaciones africanas de *A. m. scutellata* han demostrado que las ACZs son sistemas dinámicos que concentran una alta diversidad genética y que presentan diferenciación genética temporal y un tamaño efectivo de población variable, probablemente debido a un alto recambio de colonias silvestres en las cercanías (Jaffé et al. 2009). En México, a través de análisis moleculares, se encontró que existe variación estacional en la presencia de zánganos AHB y EHB en el ACZ, siendo los zánganos EHB los que salen al ACZ al final de la temporada y los AHB, al principio (Quezada-Euan et al. 2001). Al mismo tiempo, Collet et al. (2009) evaluaron la estructura genética de las ACZ y los apiarios comerciales de origen africanizado y europeo en el sur de Brasil. Utilizando marcadores microsatélites, encontraron una alta similitud genética entre las colonias del colmenar comercial y las ACZ cercanas a ellas, y diferencias en la estructura genética de las ACZ cercanas a colmenares africanizados vs. europeos. Resultados similares fueron encontrados por Mortensen & Ellis (2016) en ACZs en los Estados Unidos. En Argentina,

se han caracterizado genética y ambientalmente al menos 20 ACZs en diferentes ecorregiones (Galindo-Cardona et al. 2017; 2020).

Las diferentes razas de *A. mellifera* que pueden encontrarse en las regiones de hibridación muestran variabilidad en la morfometría alar, un carácter que varía genotípica y fenotípicamente y que, por tanto, representa una herramienta útil para la clasificación de subespecies. El uso de técnicas morfométricas que permiten obtener medidas automatizadas de los patrones de variación en las ramas venosas del ala anterior de *A. mellifera*, ha sido amplio tanto para distinguir subespecies africanas de europeas como para caracterizar los linajes evolutivos de *A. mellifera* con un alto grado de consistencia (May-Itzá et al. 2001; Francoy et al. 2008; Tofilski 2008; Miguel et al. 2011). El análisis de poblaciones presentes en áreas de hibridación natural, ya sea mediante morfometría alar y/o genética, proporciona información valiosa para la caracterización de este proceso. Por ejemplo, examinando la morfometría alar de las abejas, Porrini et al. (2022) añadieron evidencias de la existencia de un límite latitudinal para la africanización en poblaciones de *A. mellifera* en Buenos Aires, tal como habían sugerido previamente Abrahamovich et al. (2007).

Estudios pioneros en la región apícola de Argentina revelaron la existencia de seis linajes, tanto europeos como africanizados, en las poblaciones locales, siendo el haplotipo más frecuente el C1 (*A. m. ligustica*) (Sheppard et al. 1991; Abrahamovich et al. 2007; Whitfield et al. 2006). Recientemente, Agra et al. (2018) y Calfee et al. (2020) confirmaron la presencia de clinas latitudinales norte-sur entre 32º y 39º Norte y 28º y 37º Sur para el nivel de hibridación entre poblaciones de abejas europeas y africanizadas. Identificaron cuatro grupos (clusters genéticos) que se explicaban no sólo por la distribución geográfica y el grado de africanización, sino también por la influencia humana a través de las actividades apícolas. Todos los estudios mencionados se basaron en el uso de marcadores

moleculares tanto mitocondriales como nucleares como herramientas clave para la identificación de linajes mitocondriales y la caracterización de la diversidad genética.

Para explorar el grado de hibridación de las abejas en un ACZ y en un parque de fecundación de reinas como apiario de referencia, utilizamos dos técnicas complementarias: la caracterización de subespecies (morfometría geométrica del ala) y la caracterización del linaje materno (ADN mitocondrial) de abejas de Entre Ríos, Argentina. Este enfoque nos ayudó a describir los recursos genéticos locales de manera integral, ya que incluyó la contribución de los machos al proceso. Además, fue útil para estudiar la variación genética en las poblaciones presentes en el ACZ y en las colonias, lo que proporciona información valiosa sobre el estado actual de la africanización en la región y es, por lo tanto, clave para el ajuste de los programas de cría en apoyo de un desarrollo apícola sostenible.

frontiers

Materiales y métodos

Área de estudio

Este estudio se realizó en San Salvador, provincia de Entre Ríos, Argentina (31° 37' 0" Sur, 58° 30' 27" Oeste, Figura 1). Entre Ríos ocupa una gran extensión del límite oriental de la llanura pampeana, con suaves pendientes que se elevan hacia el oeste y el norte. Esta región de Argentina se caracteriza por una vegetación arbustiva, con clima templado húmedo, y condiciones templadas, aptas para el desarrollo de la actividad apícola (Ferrari et al. 2011). El régimen de precipitaciones promedio es de aproximadamente 1400 milímetros anuales. La flora con interés para los apicultores es diversa, con especies arbustivas leñosas y subarbustivas, entre las que se destacan chilcas *(Baccharis salicifolia),* chañares *(Geoffroea* sp.*),* molle *(Bumelia obtusifolia),* algarrobos *(Prosopis* sp.*),* aromitos *(Acacia caven),* tala *(Celtis tala),* ñandubay *(Prosopis affinis),* y coronillo *(Scutia buxifolia)*. La presencia de especies de interés apícola fue fundamental, ya que aportaron

polen de alta calidad proteica y néctar de composición muy variada durante siete meses (es decir, de septiembre a abril). Durante este período, la floración del eucalipto rojo *(Eucalyptus saligna)* se produce en primer lugar y la floración de las chilcas *(Baccharis salicifolia)* tiene lugar en un segundo y más importante pico de floración que finaliza en abril (Ferrari et al. 2011). En esta región los principales cultivos corresponden a maíz, soja, arroz y trigo, los cuales son producidos bajo manejo agrícola intensivo. Además, las praderas artificiales para la alimentación del ganado están compuestas por especies como alfalfa *(Medicago sativa),* trébol blanco *(Trifolium alba)* y loto *(Lotus* sp.). Muchas "malezas" de estos prados constituyen otro recurso alimenticio para las abejas melíferas y la apicultura, como las especies de cardo *(Carduus acanthoides),* cundidor *(Cirsium arvense),* cardo *(Cynara cardunculus),* asnal *(Silybum marianum)* y cardillo *(Scolymus hispanicus*) (Ferrari et al. 2011).

La búsqueda y posterior muestreo de las ACZs se llevó a cabo en el "parque de fecundación" de abejas reinas de la instalación apícola Reinas del Litoral (SENASA E-02). El parque incluía cuatro apiarios, con una distancia mínima de 1 km entre ellos (ver Figura 1). En dos de los apiarios se ubicaron 900 núcleos de apareamiento y 200-220 colonias de apoyo, organizadas por edad de las reinas. El parque de fecundacón estaba situado en un terreno llano, con alturas absolutas de 80 metros sobre el nivel del mar. El sitio se ubicó en los límites de la vegetación chaqueña y pampeana húmeda; e incluyó un fragmento de bosque de 800 a 900 hectáreas con especies naturalizadas y nativas.

Localización de las Áreas de Congregación de Drones

Para localizar las ACZ se utilizó un método basado en el rastreo directo de la ruta de entrada y salida de los zánganos del apiario de referencia. Se tomaron como referencia los colmenares 1 a 4 (Figura 1). En octubre de 2019, realizamos observaciones en la entrada de cuatro colonias del colmenar 2 y detectamos la orientación inicial de los zánganos. A

continuación, recorrimos el colmenar 2 levantando un globo de látex inflado con helio y cebado con una feromona sintética (ácido 9-hidroxi-2-enoico). Cada vez que el cebo era tocado por uno o más zánganos, marcábamos las coordenadas geográficas en un GPS (Sistema de Posicionamiento Global) (Galindo-Cardona et al. 2012; 2017; 2020). Este procedimiento se realizó tres veces al día durante tres días consecutivos y en tres puntos diferentes del potencial ACZ para confirmar los datos y determinar su tamaño. Además, una vez confirmado el ACZ, utilizamos un vehículo aéreo no tripulado (UAV), al que fijamos un cebo de feromonas, elevándolo repetidamente para capturar los zánganos (véase el punto verde en la Figura 1). La captura de los zánganos se realizó con redes entomológicas en el ACZ. Las muestras se conservaron en alcohol al 96% y se tomaron 20 zánganos al azar para su posterior análisis genético. En el laboratorio, las coordenadas geográficas tomadas con el GPS se utilizaron para cartografiar el ACZ en imágenes de satélite actualizadas (Figura 1).

frontiers

Morfometría geométrica de las alas

Para los análisis morfométricos, se tomaron muestras de abejas melíferas de 10 colonias situadas en los colmenares 1, 2 y 3 (Figura 1). El primer muestreo se realizó en otoño de 2020 en cinco colonias: P 18, P 19, V 16, V 151 y V 152. El segundo muestreo se llevó a cabo en la primavera de 2020 en cinco colonias, de las cuales dos habían sido muestreadas previamente: P19, V16, V16A, V16B, V1. Las líneas genéticas (es decir, las abejas reinas) que analizamos se seleccionaron tras dos años de evaluación y seguimiento continuo a lo largo de la temporada, que tuvo lugar de septiembre a finales de marzo. De cada colonia, se seleccionaron al azar 10 individuos y se les cortaron las alas delanteras izquierdas.

Las alas se montaron en marcos de cristal y se escanearon con una Plustek Opticfilm 8100 (LaserSoft Imaging, Kiel, Alemania) (7200 dpi). A partir de las imágenes obtenidas para cada colonia y de las correspondientes a cada subespecie pura *(A. m.*

carnica, A. m. caucasica, A. m. iberiensis, A. m. intermissa, A. m. ligustica, A. m. mellifera y A. m. scutellata, obtenidos del Morphometric Bee Data Bank de Oberursel, Alemania), se marcaron manualmente 19 puntos homólogos (Francoy et al. 2008) en tpsDIG v2.16 (Rohlf, 2010) y luego se agruparon en tpsUtil v1.4 (Rohlf, 2010).

En el software gratuito MORPHOJ v.1.07a (Klingenberg, 2011), la alineación se realizó utilizando el ajuste Procrustes para minimizar las diferencias debidas al tamaño, la posición y la rotación entre todas las configuraciones de hitos (Dryden & Mardia, 2016). Se calcularon el análisis canónico de variables (CVA) y las distancias de Mahalanobis (Klingenberg & Monteiro, 2005) para establecer el grado de similitud entre las colonias analizadas y los grupos de referencia para cada subespecie de *A. mellifera*.

Análisis genético

Para la identificación del linaje mitocondrial (es decir, europeo o africanizado) de los zánganos (muestreados en el ACZ) y de las obreras (recolectadas en el interior de cada colonia), se aplicó la técnica PCR-RFLP a la región del gen COI-COII. Esta técnica consiste en la amplificación de la región del gen y la posterior digestión del fragmento con enzimas de restricción. Se utilizaron el protocolo y los cebadores descritos por Hall & Smith (1991) y Lobo-Segura (2000), con modificaciones estandarizadas en el Laboratorio de Insectos de Importancia Agronómica (Igeaf, INTA Castelar). La extracción del ADN se realizó individualmente a partir del tórax de zánganos y obreras recolectados en el ACZ o de las colonias, respectivamente. La calidad y pureza del ADN extraído se confirmó mediante corridas electroforéticas y medición de volúmenes reducidos en el espectrofotómetro. La reacción de amplificación por PCR se llevó a cabo utilizando cebadores específicos y condiciones establecidas según Hall & Smith (1991) y Lobo-Segura (2000). La digestión de los fragmentos amplificados se llevó a cabo mediante incubación con la enzima Hinf I (Promise, Madison, MN, EE.UU.). Los fragmentos de

restricción se separaron en geles de agarosa al 3% (peso/volumen), se tiñeron con GelRed y se fotografiaron bajo luz UV. Los patrones de restricción obtenidos fueron analizados para asignar el haplotipo mitocondrial de cada muestra, considerando los patrones de referencia establecidos en la bibliografía (Agra et al., 2018). El número total de zánganos analizados del ACZ fue de 20. El número total de obreras analizadas fue de 10, una por colonia. El muestreo de abejas obreras de las 10 colonias para el análisis genético y para el análisis morfométrico se realizó simultáneamente.

Resultados

Localización de las zonas de congregación de los zánganos

Tras recorrer aproximadamente 6 km y tomando como referencia los colmenares del "parque de fecundación" (Galindo et al. 2017), encontramos tres ACZ (Figura 1). Se determinó el estado de maduración de los zánganos muestreados en las ACZs, detectando un 99% de los zánganos en estado de madurez sexual. Este valor fue superior a los encontrados para zánganos en las ACZs de Tucumán (95%) y Buenos Aires (90%) para las mismas fechas (datos no publicados). Además, realizamos evaluaciones de madurez de zánganos en colonias huérfanas (es decir, colonias productoras de celdas reinas) ubicadas a 8 km de las ACZ, y encontramos que el 20 % de esos zánganos eran inmaduros, tanto en la periferia como en el centro de la colonia.

Morfometría geométrica de las alas

Observamos que las abejas muestreadas en otoño presentaban un morfotipo africanizado, con diferentes grados de hibridación entre subespecies y mayor similitud (menores distancias de Mahalanobis) con las subespecies *A. m. caucasica, A. m. intermissa y A. m. scutellata* (Tabla 1, Figura 2). El segundo muestreo (primavera) indicó un mayor grado de hibridación entre las subespecies (Tabla 2, Figura 3), en comparación con el muestreo de

otoño y una mayor similitud con las subespecies europeas pertenecientes al linaje C (*A. m. carnica, A. m. caucasica, A. m. ligustica*).

Si analizamos los resultados del análisis morfométrico dentro de cada colonia (Tabla y Figura Suplementarias) podemos observar que aunque la mayoría de las colonias presentó mayor similitud con las subespecies *A. m. caucasica y A. m. intermissa*, existió variabilidad en relación al resto de subespecies incluidas en el análisis. En otoño, tres colonias (P18, V151, V152) mostraron menores distancias de Mahalanobis a *A. m. scutellata* que al resto de subespecies, revelando un morfotipo predominantemente africano. Por otra parte, en primavera tres colonias (P19, V16 y V16B) mostraron distancias menores a *A. m. carnica y A. m. ligustica* que a las otras subespecies y una (V16a) mostró una distancia pequeña a *A. m. mellifera*, revelando un morfotipo predominantemente europeo. En ambas estaciones (otoño y primavera), se observó un alto grado de hibridación y no hubo dominancia de una subespecie sobre las demás (es decir, las distancias de Mahalanobis fueron bastante similares).

Cuando se analizaron conjuntamente ambas estaciones, se observó que *A. m. intermissa, A. m. caucasica* y *A. m. scutellata* fueron las tres subespecies con las distancias de Mahalanobis más bajas (Tabla 3, Figura 4). Sin embargo, las distancias con el resto de subespecies no fueron mucho mayores, por lo que no podemos afirmar que el morfotipo predominante en el total de las muestras fuera únicamente africano.

Análisis genético

En el ACZ se encontraron haplotipos de origen europeo y africano (Figura 5A). El 75% de los zánganos analizados mostró un origen mitocondrial europeo (haplotipo C), siendo el C1 (65%) más frecuente y el C2J (10%), el menos frecuente (Figura 5A). El haplotipo C corresponde al linaje evolutivo de Europa del Este, donde se incluyen las abejas melíferas italianas *A. m. ligustica* y la abeja carniola, *A. m. carnica*. El 25% de los zánganos restantes

mostraron un origen mitocondrial africano (haplotipo A), con frecuencias similares para A1 (10%) y A4 (15%) (Figura 5A). Estos haplotipos se asocian generalmente con las subespecies *A. m. intermissa y A. m. scutellata*, respectivamente. En cuanto a los resultados del colmenar de referencia, el 100% de las colonias mostraron un origen mitocondrial europeo, siendo C1 el más prevalente (80%) y C2J (20%), el menos prevalente en ambas muestras (Figura 5B).

Discusión

En este estudio identificamos tres nuevas ACZs para Argentina. La caracterización de las subespecies mediante morfometría geométrica alar mostró un morfotipo de abeja variable, que incluyó tanto a subespecies europeas como a africanas, y diferentes grados de hibridación. En este sentido, el enfoque morfométrico permitió revelar una representación considerable de híbridos en las colonias y su aplicación es necesaria para describir si el patrón en los zánganos en el ACZ es similar. La caracterización del linaje materno (ADN mitocondrial) en zánganos recolectados en un ACZ evidenció la presencia de haplotipos tanto de origen africano como europeo, con mayor prevalencia de estos últimos. Congruentemente, detectamos la presencia exclusiva de linajes europeos (C1 y C2J) en las abejas del colmenar de referencia.

Localización de las áreas de congregación de zánganos y caracterización del colmenar de referencia

Las ACZs localizadas en este estudio presentaban características paisajísticas similares a otras ACZs en Argentina, tales como estructura de vegetación simple (i.e., pastizales y áreas abiertas) y terreno ondulado con baja pendiente.

El alto porcentaje de zánganos maduros encontrados en el ACZ podría deberse a que el muestreo se realizó al inicio de la época reproductiva, cuando la población es

reducida debido a la falta de recursos florales. Otra posibilidad es que las condiciones climáticas previas a la época de apareamiento aceleren el desarrollo de colonias productoras de zánganos (observación personal, LL y FM). En las colonias productoras de celdas de reinas también encontramos un alto porcentaje de madurez de zánganos. Los apicultores que crían reinas han observado entradas masivas de zánganos en este tipo de colonias. Una explicación plausible de este patrón es que los zánganos emiten feromonas que atraen a otros zánganos maduros (Brandstaetter et al. 2014, Villar et al. 2017) de colonias cercanas o pueden proceder de colonias huérfanas y entrar libremente para alimentarse (Galindo-Cardona et al. 2015). Además, las reinas vírgenes emiten sonidos ("cuac") tras la emergencia para avisar a las obreras de que están a punto de abandonar la colonia e inhibir la salida de las otras reinas (Ramsey et al. 2018). Este sonido podría generar aglomeraciones de zánganos alrededor de las celdas de las reinas que están próximas a emerger (observación personal, LL y FM). Observamos que las celdas de reinas estaban rodeadas por varios zánganos y que estas colonias presentaban un elevado número y diversidad fenotípica de zánganos maduros en su mayoría. Este comportamiento sugiere que la presencia de los zánganos podría ser un estímulo para el desarrollo de las reinas que aún están dentro de sus celdas, hipótesis que debe ser comprobada experimentalmente. Concluimos que el inicio de la temporada de apareamiento es una ventana temporal apropiada para gestionar la reproducción de reinas en las ACZ seleccionando zánganos con las características deseadas.

Morfometría geométrica alar

La variabilidad encontrada en los morfotipos que analizamos es consistente con los resultados obtenidos previamente en poblaciones manejadas en Argentina (Bianchi et al. 2021; Porrini et al. 2022). En las zonas de transición entre poblaciones europeas y africanizadas, donde el flujo génico es extenso, generalmente se observan diferencias a

frontiers

nivel mitocondrial, nuclear y morfométrico porque representan zonas híbridas (Whitfield et al. 2006; Sheppard et al. 1991). Las colonias de estas zonas de transición presentan a menudo rasgos africanos, incluso en áreas donde persisten proporciones relativamente altas de alelos europeos. El predominio de alelos africanos no tiene por qué provocar la pérdida de marcadores europeos, sino que contribuye a la conservación del fenotipo africano (Scott Schneider et al. 2004). Por otro lado, aunque la alta diversidad intra colonial que encontramos podría estar confiriendo a las colonias un mayor grado de resistencia a ciertos patógenos y/o una mayor productividad en términos adaptativos (Desai & Currie 2015; Oldroyd et al. 1992; Mattila & Seeley 2007), creemos que es necesario evaluar en el futuro otros factores, como el manejo de la población, el tipo de multiplicación utilizada y el flujo de recursos en diferentes regiones y épocas del año, para avanzar en dicha hipótesis.

Los resultados más interesantes al comparar la forma de las alas se observan a nivel de colonia. La elevada variación intra colonia aquí detectada debe seguir evaluándose en diferentes regiones y escalas temporales para proporcionar a los criadores de reinas una rápida caracterización genética de sus poblaciones de cara a la selección y multiplicación. Como perspectivas futuras, creemos que es importante explorar la variabilidad de los morfotipos de zánganos en las ACZ. Una limitación de este tipo de análisis es que, a diferencia de las abejas obreras, no se dispone de un banco de imágenes de alas de zánganos como referencia para las distintas subespecies de *A. mellifera*. Sin embargo, es posible comparar el morfotipo de los zánganos de las áreas de congregación con los zánganos de las colonias manejadas cercanas (May-Itzá et al. 2001).

Análisis genético

Nuestros resultados indicaron una gran homogeneidad en el origen mitocondrial de las colonias del colmenar de referencia, ya que todas presentaban el haplotipo mitocondrial europeo. Nuestros resultados son consistentes con el hecho de que la apicultura en esta

zona de la provincia de Entre Ríos se realiza principalmente con materiales de origen europeo, ya que los apicultores mantienen el uso de esta genética a través de la implementación de estrategias de manejo como programas de cría de reinas y abejas (Agra et al. 2018). En las ACZ detectamos un patrón genético similar al observado en Buenos Aires, y contrastante con lo observado para la provincia de Tucumán, donde las ACZ tuvieron una alta representación de zánganos de origen africano (Galindo-Cardona et al. 2020). Si bien el patrón encontrado en Entre Ríos indicaba una alta proporción de zánganos de origen europeo, fue posible detectar un 25% de haplotipos mitocondriales africanos (A1 y A4); estos valores son significativamente superiores a los observados en el ACZ de Buenos Aires (3%; Galindo-Cardona et al. 2020). Los estudios sobre la proporción de linajes africanos vs. europeos dentro de las ACZs en otras regiones del mundo se limitan al de Mortensen y Ellis (2016) en una región no hibridizada de EEUU, quienes mostraron que esta proporción favorecía a los linajes africanos cuando aumentaba la distancia a las colonias europeas manejadas. A pesar del impacto que la apicultura puede tener en la dinámica y genética de las abejas en el ACZ, estos sitios mantienen una mayor diversidad de haplotipos en comparación con sus apiarios de referencia de origen, lo que sugiere la importancia de protegerlos como reservorios de variabilidad genética para las poblaciones de la especie.

En México existe un reporte de un aislamiento estacional parcial en la reproducción de zánganos de origen africano y europeo en las proximidades de un ACZ (Quezada-Euan et al. 2001). Nuestros hallazgos detectaron que, tanto en el ACZ como en el colmenar de referencia, los haplotipos EHB predominaron al principio de la temporada. Una posible variación estacional en la frecuencia de haplotipos debe evaluarse más a fondo en Argentina y compararse con el patrón encontrado en el hemisferio norte.

Mortensen y Ellis (2016) demostraron que la proporción de zánganos de un origen genético concreto en las ACZ puede verse afectada por la "inundación" de zánganos. Esta

frontiers

técnica podría tener una gran influencia en los programas de cría selectiva, ya que permitiría controlar parcialmente la contribución paterna en las poblaciones reproductoras de abejas melíferas (Guzmán-Novoa y Page, 1999, Mortensen y Ellis, 2016). En este sentido, la relativamente baja abundancia de haplotipos africanos en el ACZ en este estudio podría ser producto de la alta influencia del colmenar de referencia y otros colmenares comerciales circundantes, que multiplican genes de origen europeo casi exclusivamente. Por lo tanto, es clave diseñar estrategias que monitoreen adecuadamente la diversidad genética de las abejas en las ACZ en diferentes momentos de la estación de apareamiento, tanto para su aplicación a la toma de decisiones y acciones de gestión apícola, como para responder a cuestiones eco-evolutivas como la comprensión de los mecanismos que conducen a la cooperación de poblaciones de abejas de origen diverso (por ejemplo, africanizadas, europeas, ecotipos locales) en la formación de un ACZ.

La convergencia de AHB y EHB en los lugares de hibridación ha sido un tema de gran interés en la investigación del flujo genético en *Apis melifera*. Métodos más sofisticados que los utilizados aquí, como microsatélites o SNPs, mostraron una mejor resolución genética en estos sitios (Agra et al. 2018 y Calfee et al. 2020). Es importante destacar, sin embargo, que utilizando técnicas moleculares y morfométricas menos costosas se pueden obtener resultados similares (Lobo et al. 1989, Moritz & Meusel 1992, Lobo 1995, Quezada-Euan & Medina 1998). En conclusión, nuestros resultados apoyan la existencia de poblaciones híbridas basadas en un morfotipo variable que comprende tanto subespecies europeas como africanas, y en el linaje mitocondrial. Las áreas de congregación de zánganos representan interesantes zonas de estudio que permiten evaluar poblaciones de abejas localmente adaptadas y su evolución natural a lo largo del tiempo. Estos ACZ desempeñan un papel crucial en la supervivencia de las abejas, que participan en la polinización y son esenciales para nuestra seguridad alimentaria. Además, destacamos la necesidad de proteger globalmente estas áreas de apareamiento, por su valor

como reservorios genéticos (Barton & Hewitt 1985) y de aprovecharlas para la evaluación de materiales vivos de la región.

Agradecimientos

Reinas del Litoral (habilitación SENASA E-02) y a la municipalidad de San Salvador de Entre Ríos, quienes brindaron asistencia de campo y facilidades de comunicación, y organizaron reuniones con funcionarios de la municipalidad y el público. Agradecemos al Dr. Tugrul Giray por sus contribuciones al mejoramiento del artículo. Agradecemos a dos revisores anónimos sus valiosos comentarios y sugerencias, que contribuyeron a mejorar el artículo. Los fondos para insumos y transporte al lugar de trabajo fueron obtenidos del PIP2015-0517CO y PIP2020-2908CO (AGC y ACS), del CONICET.

Referencias

Abrahamovich, A., N. Díaz and M. Lucia. 2007. Identificación de las “abejas sociales” del género *Bombus* (Hymenoptera, Apidae) presentes en la Argentina: clave pictórica, diagnosis, distribución geográfica y asociaciones florales. *Revista de la Facultad de Agronomía*, La Plata, 106(2), 165-176.

Agra, M.N., C.A. Conte, P.M. Corva, J.L. Cladera, S.B. Lanzavecchia and M.A. Palacio. 2018 Molecular characterization of *Apis mellifera* colonies from Argentina: genotypic admixture associated with ecoclimatic regions and apicultural activities. *Entomologia Experimentalis et Applicata*, 166, 724-738.

Barton, N.H. and G.M. Hewitt. 1985. Analysis of hybrid zones. *Annual Review of Ecology and Systematics. 16*, 113–148. https://doi.org/10.1146/annurev.es.16.110185.000553

Bastin, Florian; Cholé, Hanna; Lafon, Grégory; Sandoz, Jean-Christophe. 2017. Virgin queen attraction toward males in honey bees. *Scientific Reports, 7(1), 6293–* .doi:10.1038/s41598-017-06241-9

Bianchi, E., M.N. Agra, C. García, G. Gennari, L. Maldonado, G.A. Rodríguez, M.A. Palacio, A.C. Scannapieco and S.B. Lanzavecchia. (2021). Defensive Behavior and Morphometric Variation in Apis mellifera Colonies From Two Different Agro-Ecological Zones of North-Western Argentina. *Frontiers in Ecology and Evolution*, 585.

Bortolotti, L. and C. Costa. 2014. Chemical Communication in the Honey Bee Society. In: Mucignat-Caretta C, editor. Neurobiology of Chemical Communication. Boca Raton (FL): CRC Press/Taylor & Francis; Chapter 5. PMID: 24830041.

Brandstaetter, A.S., F. Bastin and J.C. Sandoz. 2014. Honeybee drones are attracted by groups of consexuals in a walking simulator. *J Exp Biol.*;217(Pt 8):1278-85. doi: 10.1242/jeb.094292. PMID: 24436379.

Brutscher, L.M., B. Baer and E.L. Niño. 2019. Putative Drone Copulation Factors Regulating Honey Bee (*Apis mellifera*) Queen Reproduction and Health: A Review. *Insects,* 10, 1: 8. https://doi.org/10.3390/insects1001000

Calfee, E., M.N. Agra, M.A. Palacio, S.R. Ramírez and G. Coop. 2020. Selection and hybridization shaped the rapid spread of African honey bee ancestry in the Americas. *PLoS Genet,* 16(10): e1009038.

Collet, T., A.S. Cristino, C.F. Quiroga, A.E. Soares and M.A. Del Lama. 2009. Genetic structure of drone congregation areas of Africanized honeybees in southern Brazil. *Genetics and molecular biology*, *32*(4), 857–863. https://doi.org/10.1590/S1415-47572009005000083.

Desai, S.D. and R.W. Currie. 2015. Genetic diversity within honey bee colonies affects pathogen load and relative virus levels in honey bees, Apis mellifera L. *Behavioral ecology and sociobiology*, *69*(9), 1527-1541.

frontiers

Dryden, I.L. and K.V. Mardia. 2016. Statistical Shape Analysis, with Applications in R (2nd ed.). Chichester: Wiley.

Endler, J.A. 1986. Natural Selection in the Wild. (MPB-21), Volume 21. Princeton University Press. https://doi.org/10.2307/j.ctvx5w9v9

Ferrari, C.A., R. Currao and J. Folgar. 2011. La Apicultura Argentina y sus regiones. Una visión panorámica; coordinado por Ramiro Otero y Jorge A Collía. - 1a ed. - Buenos Aires: Consejo Federal de Inversiones. 200 p. : il.; 29x21 cm. - (Aportes conceptuales y metodológicos)

Francoy, T.M., D. Wittmann, M. Drauschke, S. Müller, V. Steinhage, M.A.F. Bezerra-Laure, D.D. Jong, and L.S. Gonçalves. 2008. Identification of Africanized honey bees through wing morphometrics: two fast and efficient procedures. Apidologie, 39(5), 488– 494. doi:10.1051/apido:2008028

Galindo-Cardona, A., A.C. Monmany-Garzia, R. Moreno-Jackson, C. Rivera-Rivera, C. Huertas-Dones, L. Caicedo-Quiroga and T. Giray. 2012. Landscape analyses of drone congregation areas of the honey bee *Apis mellifera*. *Journal of Insect Science.* 12:104.

Galindo-Cardona, A., J.P. Acevedo-Gonzalez, B. Rivera-Marchand and T. Giray. 2013. Genetic structure of the gentle Africanized honey bee population (gAHB) in Puerto Rico. *BMC Genetics*, *14*. https://doi.org/10.1186/1471-2156-14-65

Galindo-Cardona, A., A.C. Monmany, G. Diaz and T. Giray. 2015. *A* Landscape Analysis to Understand Orientation of Honey Bee (Hymenoptera: Apidae) Drones in Puerto Rico. *Environmental Entomology, 44(4), 1139.1–1148.*doi:10.1093/ee/nvv099

Galindo-Cardona, A., B. Quiroga, E. Bianchi and M.M. Ayup. 2017. Primer reporte de un Área de Congregación de Zánganos de *Apis mellifera* (Hymenoptera: Apidae) de Argentina. *Revista de la Sociedad Entomológica Argentina.* 76: 1-4. doi.org/10.25085/rsea.761207

Galindo-Cardona, A., A.C. Scannapieco, R. Russo, K. Escalante, M. Geria, N. Lepori, M.M. Ayup, I. Muntaabski, M.C. Liendo, L. Landi, T. Giray and A.C. Monmany-Garzia. 2020. *Varroa destructor* Parasitism and Genetic Variability at Honey Bee (*Apis mellifera*) Drone Congregation Areas and Their Associations With Environmental Variables in Argentina. *Frontiers in Ecology and Evolution,* 8:590345. doi.org/10.3389/fevo.2020.590345

Guzmán-Novoa, E. and Page, R. E. 1999. Selective Breeding of Honey Bees (Hymenoptera: Apidae) in Africanized Areas. *Journal of Economic Entomology, 92(3), 521–525.* doi:10.1093/jee/92.3.521

Hall, H.G., Smith D.R. 1991. Distinguishing African and European honeybee matrilines using amplified mitochondrial DNA. *Proc. Natl. Acad. Sci.*, 88: 4548-4552.

Harrison, R.G. 1990. Hybrid zones: windows on evolutionary process. In: Futuyma D, Antonovics J, editors. Oxford surveys in evolutionary biology. Vol. 7. New York: Oxford University Press. p. 69–128

Jaffé, R., V. Dietemann, R.M. Crewe and R.F.A. Moritz. 2009. Temporal variation in the genetic structure of a drone congregation area: an insight into the population dynamics of wild African honeybees (*Apis mellifera scutellata*). *Molecular Ecology*, 18: 1511-1522. https://doi.org/10.1111/j.1365-294X.2009.04143.x

Klingenberg, C.P. and L.R. Monteiro. 2005. Distances and directions in multidimensional shape spaces: Implications for morphometric applications. *Systematic Biology*, 54(4), 678–688. doi:10.1080/10635150590947258

Klingenberg, C.P. 2011. MorphoJ: An integrated software package for geometric morphometrics. *Molecular Ecology Resources*, 11(2), 353-357. doi:10.1111/j.1755-0998.2010.02924.x

Koeniger, N., G. Koeniger, M. Gries and S. Tingek. 2005. Drone competition at drone congregation areas in four *Apis* species. *Apidologie*, 36(2), pp.211-221. hal- 00892135.

frontiers

Lobo Segura, J.A. 1995. Morphometric, isozymic and mitochondrial variability of Africanized honeybees in Costa Rica, *Heredity* 75, 133-141.

Lobo Segura, J.A. Del Lama M.A.,MestrinerM.A. 1989. Population differentiation and racial admixture in the Africanized honeybee (*Apis mellifera* L.), *Evolution*, 43,794 - 802.

Lobo Segura, J. A. 2000. Highly polymorphic DNA markers in Africanized honey bee populations in Costa Rica. *Genetics and Molecular Biology*, 23(2), 317–322. doi:10.1590/S1415- 47572000000200013

Loper, G.M., O.R. Wolf and J.R. Taylor. 1993. Radar detection of drones responding to honeybee queen pheromone. *J. Chem. Ecol*. 19, 1929–1937.

May-Itzá, W.J., J J G Quezada-Euán, L Iuit & C M Echazarreta. 2001. Do morphometrics and allozymes reliably distinguish Africanized and European *Apis mellifera* drones in subtropical Mexico?, *Journal of Apicultural Research*, 40:1, 17-23, DOI: 10.1080/00218839.2001.11101044

Mattila, H. R. and T.D. Seeley. 2007. Genetic diversity in honey bee colonies enhances productivity and fitness. *Science*, *317*(5836), 362-364.

Miguel, I., M. Baylac, M. Iriondo, C. Manzano, L. Garnery and A. Estonba. 2011. Both geometric morphometric and microsatellite data consistently support the differentiation of the *Apis mellifera* M evolutionary branch. *Apidologie* 42: 150-161.

Moritz R.F.A. and M.J. Meusel. 1992. Mitochondrial gene frequencies in Africanized honey bees (*Apis mellifera* L.) theoretical model and empirical evidence, *J. Evol. Biol*. 5, 71-81.

Mortensen, A.N. and J.D. Ellis. 2016. Managed European-derived honey bee, *Apis mellifera* sspp, colonies reduce African-matriline honey bee, *A. m. scutellata*, drones at regional mating congregations. *PLoS One,* 11:e0161331.

Mortensen, A.N., C.J. Jack and J.D. Ellis. 2018. The discovery of *Varroa destructor* on drone honey bees, *Apis mellifera*, at drone congregation areas. *Parasitol. Res*., 117, 3337-3339

Oertel, E. 1956. Observations on the Flight of Drone Honey Bees. *Annals of The Entomological Society of America, 49*, 497-500.

Oldroyd, B.P., T.E. Rinderer, J.R. Harbo and S.M. Buco. 1992. Effects of intracolonial genetic diversity on honey bee (Hymenoptera: Apidae) colony performance. *Annals of the Entomological Society of America*, *85*(3), 335-343.

Oxley, P.R. and B.P. Oldroyd, 2010. The Genetic Architecture of Honeybee Breeding. In Stephen J. Simpson and Jérôme Casas, editors: Advances in Insect Physiology, Vol. 39, Burlington: Academic Press, pp. 83-118.ISBN: 978-0-12-381387-9

Porrini, L. P., Quintana, S., Brasesco, C., Maggi, M. D., Porrini, M. P., Garrido, M. P., and Eguaras, M. J. 2022. Current genetic diversity of managed and commercially produced Apis mellifera colonies in Argentina inferred by wing geometric morphometrics and COI-COII mtDNA locus. *Apidologie*, *53*(5), 1-17.

Quezada-Euan J.J.G., and L.M. Medina. 1998. Hybridization between European and Africanized honeybees (*Apis mellifera* L.) in tropical Yucatan, Mexico. I. Morphometric changes in feral and managed colonies. *Apidologie*, 29 (6), pp.555-568. hal-00891557

frontiers

Quezada-Euan J.J.G., De J. May-Itza W. 2001. Partial seasonal isolation of African and European-derived *Apis mellifera* (Hymenoptera: Apidae) drones at congregation areas from subtropical Mexico. *Ann. Entomol. Soc. Am.* 94(4):540–44

Ramsey, M., M. Bencsik, M. Newton. 2018. Extensive Vibrational Characterisation and Long-Term Monitoring of Honeybee Dorso-Ventral Abdominal Vibration signals. Scientific Reports. 8. 10.1038/s41598-018-32931-z.

Rohlf, F.J. 2010a. tps-DIG, Digitize Landmarks and Outlines, version 2.16. Department of Ecology and Evolution, State University of New York at Stony Brook, New-York.

Rohlf, F.J. 2010b. tps-UTIL, File Utility Program, version 1.46. Department of Ecology and Evolution, State University of New York at Stony Brook, New-York.

Ruttner, F. (1966). The life and flight activity of drones. Bee World, 47(3), 93-100.

Schneider, S., G. DeGrandi-Hoffman and D.R. Smith. 2004. The African honey bee: Factors contributing to a successful biological invasion. Annual Review of Entomology, 49(1), 351–376. doi:10.1146/annurev.ento.49.061802.123359.

Sheppard W. 1989. A history of the introduction of honey bee races into the United States: Part I and II. *Amer. Bee Journal,* 129, 617-619, 664-667

Sheppard, W.S., A.E.E. Soares, D. De Jong and H. Shimanuki. 1991. Hybrid status of honey bee populations near the historic origin of Africanization in Brazil. *Apidologie*, 22, 643-652.

Strang, G.E. 1970. A study of honey bee drone attraction in mating response. *J. Econ. Entomol*., 63, pp. 641-645.

Tatsuta, H., K.H. Takahashi and Y. Sakamaki. 2018. Geometric morphometrics in entomology: Basics and applications. *Entomological Science*, *21*(2), 164-184.

frontiers

Tofilsky, A. 2008. Using geometric morphometrics and standard morphometry to discriminant three honeybee subspecies. *Apidologie* 38:538-563.

Tribe, G.D. 1982. Drone mating assemblies. *S. Afr. Bee J.* 54 99–100; 103–117.

Villar, G., M.D. Wolfson, A. Hefetz and C.M. Grozinger. 2018. Evaluating the role of drone-produced chemical signals in mediating social interactions in honey bees (*Apis mellifera)*. *J Chem Ecol.* 44, 1–8. https://doi.org/10.1007/s10886-017-0912-2

von Frisch, K. 1957. La vida de las abejas. Colección Labor. Rodriguez Editores. 224 pp.

Whitfield, C.W., S.K. Behura, S.H. Berlocher, A.G. Clark, J.S. Johnston, W.S. Sheppard, D.R. Smith,

A.V. Suarez, D. Weaver and N.D. Tsutsui. 2006. Thrice Out of Africa: Ancient and Recent Expansions of the Honey Bee, *Apis mellifera*. *Science*, 314, 642–645.

Withrow, J.M. and D.R. Tarpy. 2018. Cryptic. “royal” subfamilies in honey bee (*Apis mellifera*). colonies. *PLoS ONE* 13(7): e0199124.

Winston, M.L. 1987. The biology of the honey bee, Harvard University Press, Cambridge, Massachu- setts.

Zmarlicki, C., and R.A. Morse. 1963. Drone congregation areas. *Journal of Apicultural Research*, 2(1), 64-66.

Tabla 1 - Distancias de Mahalanobis. Muestreo 1- Distancias genéticas de las muestras a cada subespecie pura de *Apis mellifera* (*A. m. carnica, A. m. caucasica, A. m. iberiensis, A. m. intermissa, A. m. ligustica, A. m. mellifera,* and *A. m. scutellata*). * indica las distancias genéticas más pequeñas.

Subespecie	Muestreo 1 - 07/03/2020
A. m. carnica	5,945
A. m. caucasica	4,809*
A. m. iberiensis	6,244
A. m. intermissa	4,679*
A. m. ligustica	5,772
A. m. mellifera	5,419
A. m. scutellata	5,027*

frontiers

Tabla 2 - Distancias de Mahalanobis. Muestreo 2- Distancias genéticas de las muestras a cada subespecie pura de *Apis mellifera* (*A. m. carnica, A. m. caucasica, A. m. iberiensis, A. m. intermissa, A. m. ligustica, A. m. mellifera,* and *A. m. scutellata*). * indica las distancias genéticas más pequeñas.

Subespecie	Muestreo 2 - 15/12/2020
A. m. carnica	5,256*
A. m. caucasica	5,320*
A. m. iberiensis	6,425
A. m. intermissa	5,451*
A. m. ligustica	5,450*
A. m. mellifera	5,850
A. m. scutellata	5,973

frontiers

Tabla 3 - Distancias de Mahalanobis. Muestreos 1 y 2- Distancias genéticas de las muestras a cada subespecie pura de *Apis mellifera* (*A. m. carnica, A. m. caucasica, A. m. iberiensis, A. m. intermissa, A. m. ligustica, A. m. mellifera,* and *A. m. scutellata*). * indica las distancias genéticas más pequeñas.

Subespecie	Muestreos 1 y 2 - 07/03/2020 y 15/12/2020
A. m. carnica	5,187
A. m. caucasica	4,757*
A. m. iberiensis	6,159
A. m. intermissa	4,683*
A. m. ligustica	5,231
A. m. mellifera	5,365
A. m. scutellata	5,072*

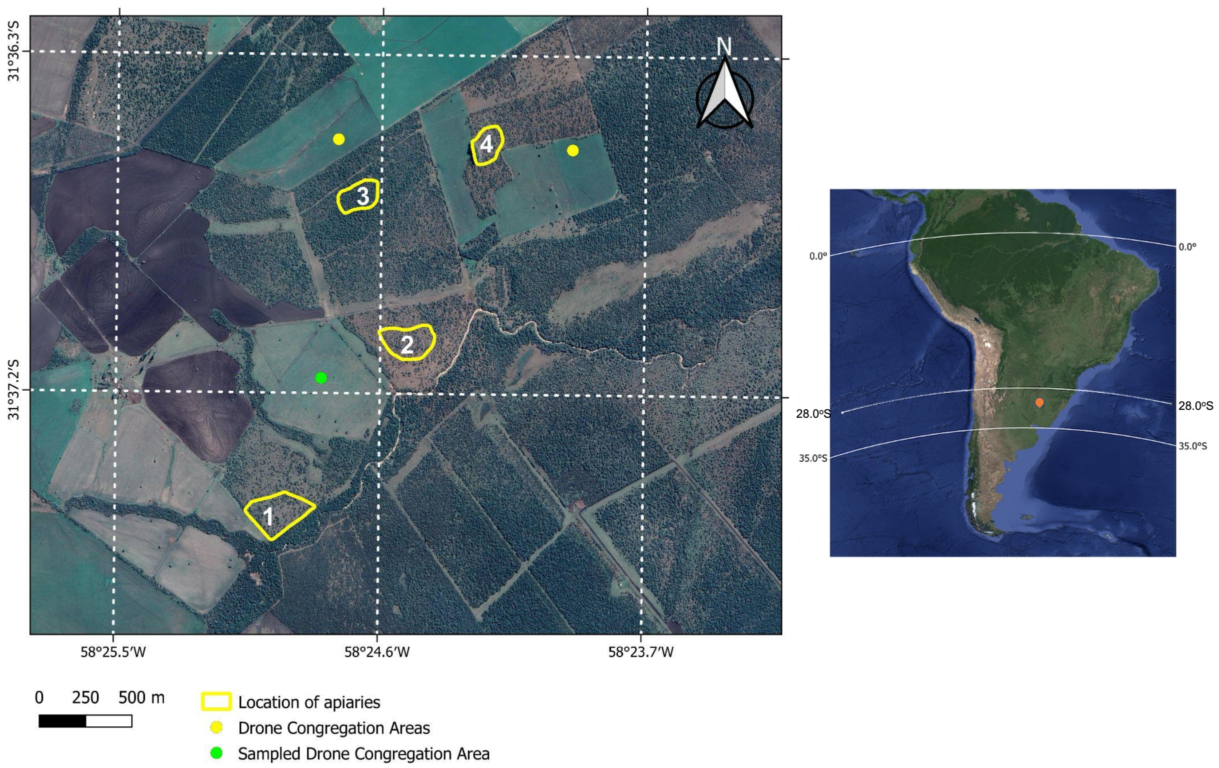

Figura 1. Imágenes de satélite de Google actualizadas de la zona de estudio (izquierda) y de Sudamérica (derecha). En la imagen de Sudamérica, el área de estudio está marcada con un punto naranja; la zona de hibridación se produce entre los 28° S y los 35° S en Argentina. La imagen del área de estudio (izquierda) muestra las tres áreas de congregación de zánganos (ACZ) y los cuatro apiarios incluidos en este estudio y ubicados cerca de la ciudad de San Salvador, Entre Ríos. El ACZ de donde se recolectaron las muestras para este estudio está marcada en verde. Las muestras para los análisis de morfometría alar se tomaron de los apiarios 1, 2 y 3. Los apiarios 1 a 4 se utilizaron para observar las rutas de salida de los zánganos a las ACZ.

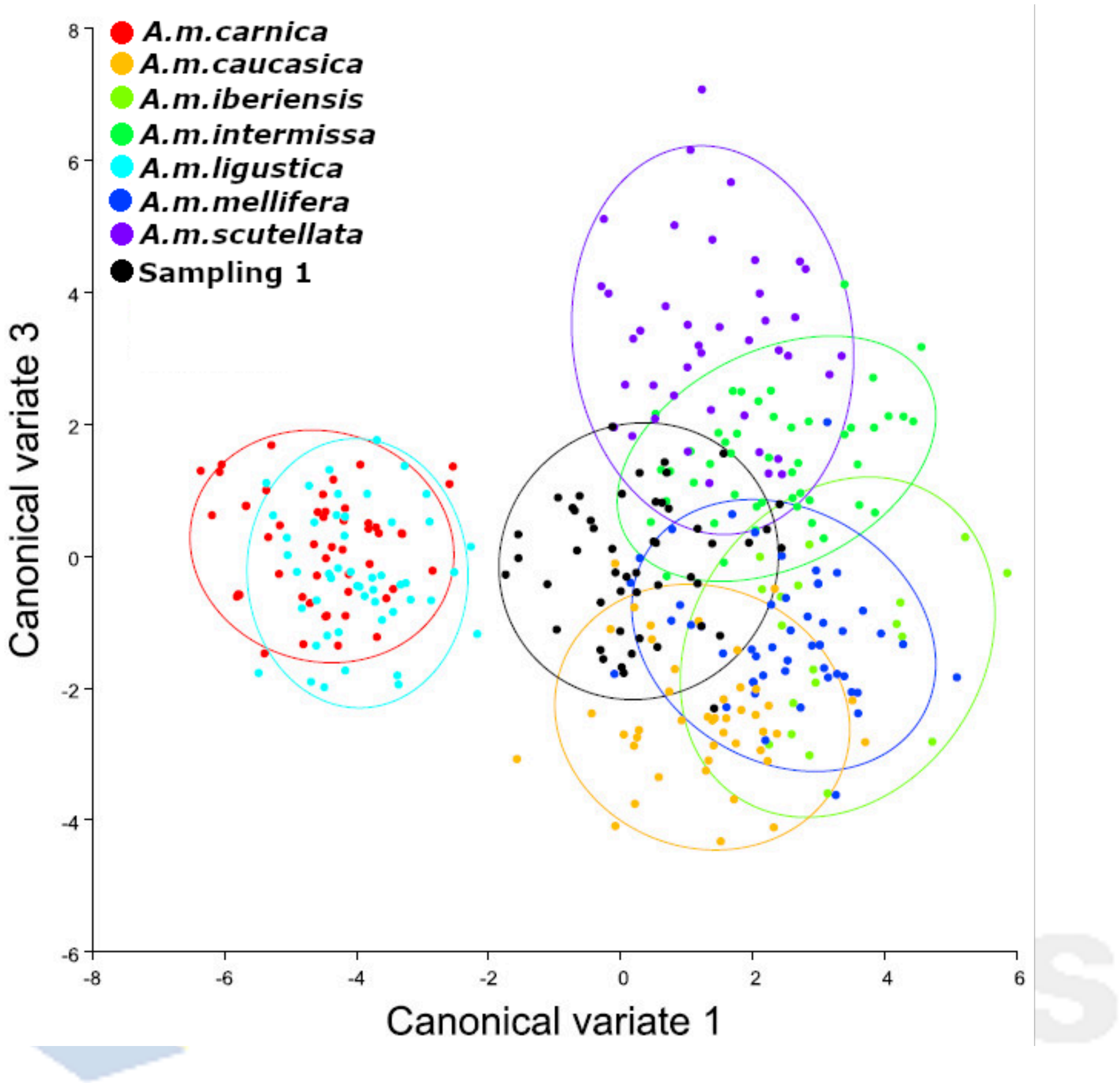

Figura 2. Análisis de variables canónicas. Muestreo 1 - Comparación con muestras de referencia de subespecies de *A. mellifera (A. m. carnica* = rojo; *A. m. caucasica* = naranja; *A. m. iberiensis* = verde claro; *A. m. intermissa*= verde; *A. m. ligustica* = turquesa; *A. m. mellifera* = azul y *A. m. scutellata* = púrpura). La muestra analizada está representada en negro (Reinas del Litoral). Se utilizaron 10 individuos (alas) por colonia y 50 individuos (alas) por grupo de referencia para cada subespecie, excepto para *A. m. iberiensis*, que fueron 20.

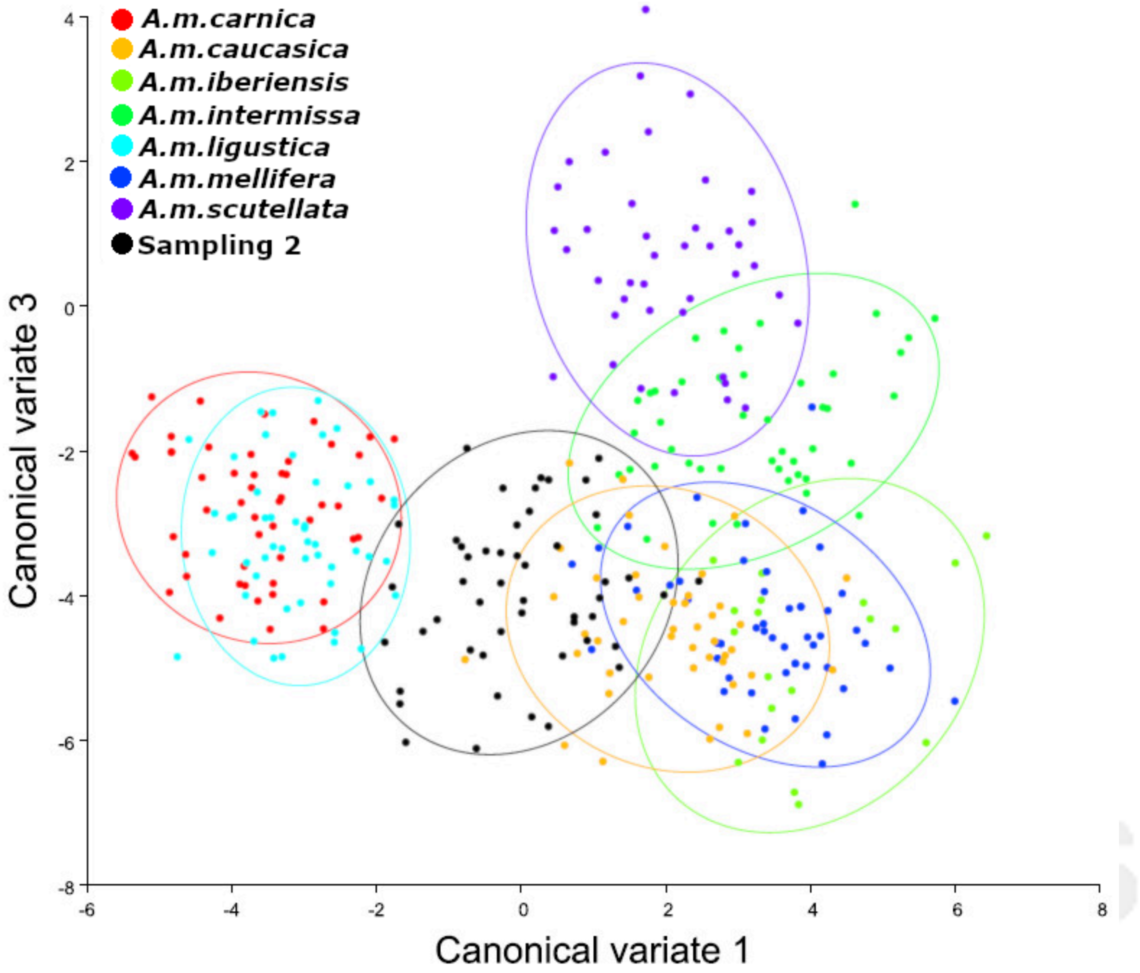

Figura 3. Análisis de variables canónicas. Muestreo 2 - Comparación con muestras de referencia de subespecies de *A. mellifera* (*A. m. carnica* = rojo; *A. m. caucasica* = naranja; *A. m. iberiensis* = verde claro; *A. m. intermissa*= verde; *A. m. ligustica* = turquesa; *A. m. mellifera* = azul y *A. m. scutellata* = púrpura). La muestra analizada está representada en negro (Reinas del Litoral). Se utilizaron 10 individuos (alas) por colonia y 50 individuos (alas) por grupo de referencia para cada subespecie, excepto para *A. m. iberiensis*, que fueron 20.

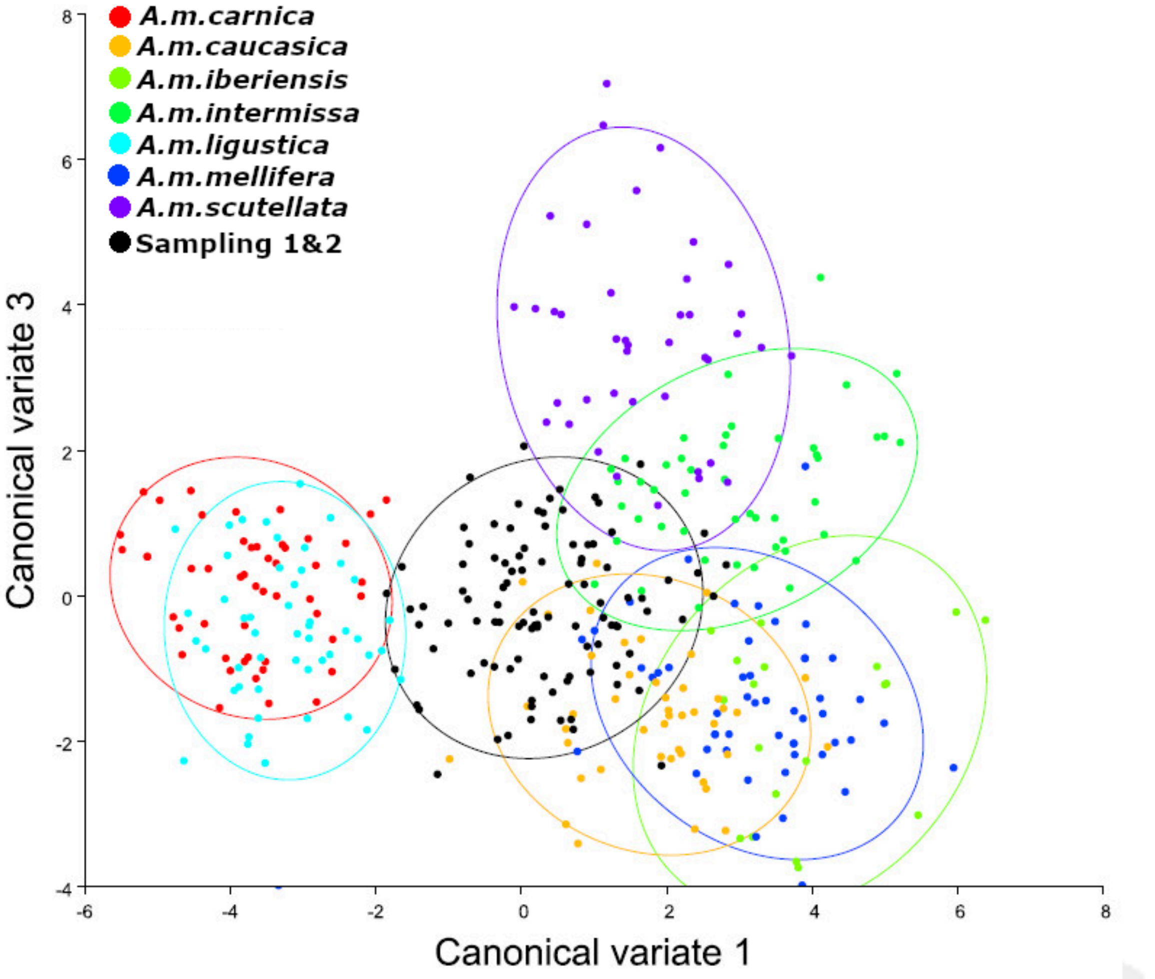

Figura 4. Análisis de Variables Canónicas (CVA). Las dos primeras variantes canónicas ilustran la variación de la forma alar en los muestreos 1 y 2 (color negro), en comparación con muestras de referencia de subespecies de *A. mellifera* (*A. m. carnica* = rojo; *A. m. caucasica* = naranja; *A. m. iberiensis* = verde claro; *A. m. intermissa*= verde; *A. m. ligustica* = turquesa; *A. m. mellifera* = azul y *A. m. scutellata* = morado). Cada marcador (puntos) representa las puntuaciones medias de cada colonia. Las elipses representan intervalos de confianza del 95% alrededor del centroide de cada grupo de datos. Se utilizaron 10 individuos (alas) por colonia y 50 individuos (alas) por grupo de referencia para cada subespecie, excepto para *A. m. iberiensis*, que fueron 20.

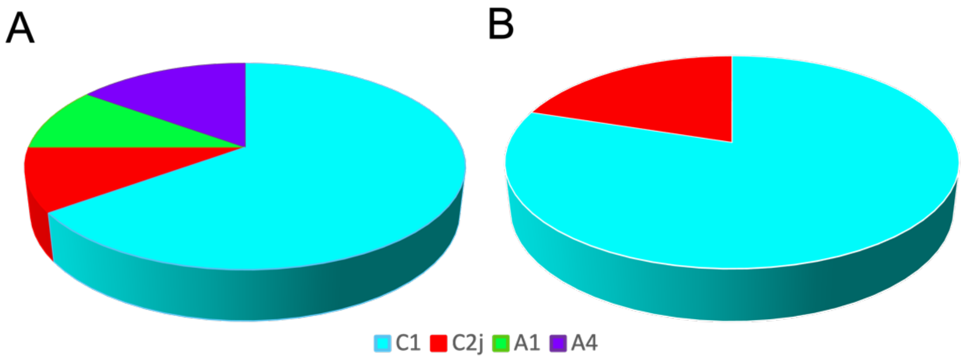

Figura 5. Porcentajes de haplotipos mitocondriales detectados en los zánganos de *Apis mellifera* muestreados en el ACZ (A) y en las obreras de las 10 colonias del colmenar de referencia (B). C1 y C2j corresponden a haplotipos europeos, mientras que A1 y A4 corresponden a haplotipos de origen africano. Se utilizaron 20 individuos (zánganos) por ACZ muestreada y un individuo (obrera) por colmena de referencia.

Tabla suplementaria 1 - Distancias de Mahalanobis. Distancia genética de las muestras a cada subespecie pura de *A. mellifera* (*A. m. carnica, A. m. caucasica, A. m. iberiensis, A. m. intermissa, A. m. ligustica, A. m. mellifera y A. m. scutellata*). * indica las distancias genéticas más pequeñas.

Subespecies	07/03/2020 Muestreos					15/12/2020 Muestreos				
	P18	P19	V16	V151	V152	V16A	P19	V1	V16	V16B
A. m. carnica	7,052	6,186	7,092	5,470	7,403	6,484	4,946*	6,747	5,413*	6,267*
A. m. caucasica	5,558*	5,659*	5,564*	4,967*	6,152*	6,064*	5,414*	5,644*	6,609	5,871*
A. m. iberiensis	7,037	6,881	6,920	6,279	7,082	6,676	6,926	6,341	7,592	7,234
A. m intermissa	5,018*	5,925*	6,027*	4,461*	6,177*	6,029*	5,546*	6,065*	6,725	6,420*
A. m. ligustica	6,955	5,979*	6,895	5,476	6,976	6,691	5,179*	6,431	5,973*	6,456*
A. m. mellifera	5,896*	5,918*	6,519*	5,300	7,158	5,780*	6,174	6,353	6,851	6,775
A. m. scutellata	5,321*	6,322	7,109	4,596*	6,230*	6,824	6,063	6,382	7,496	6,816

frontiers

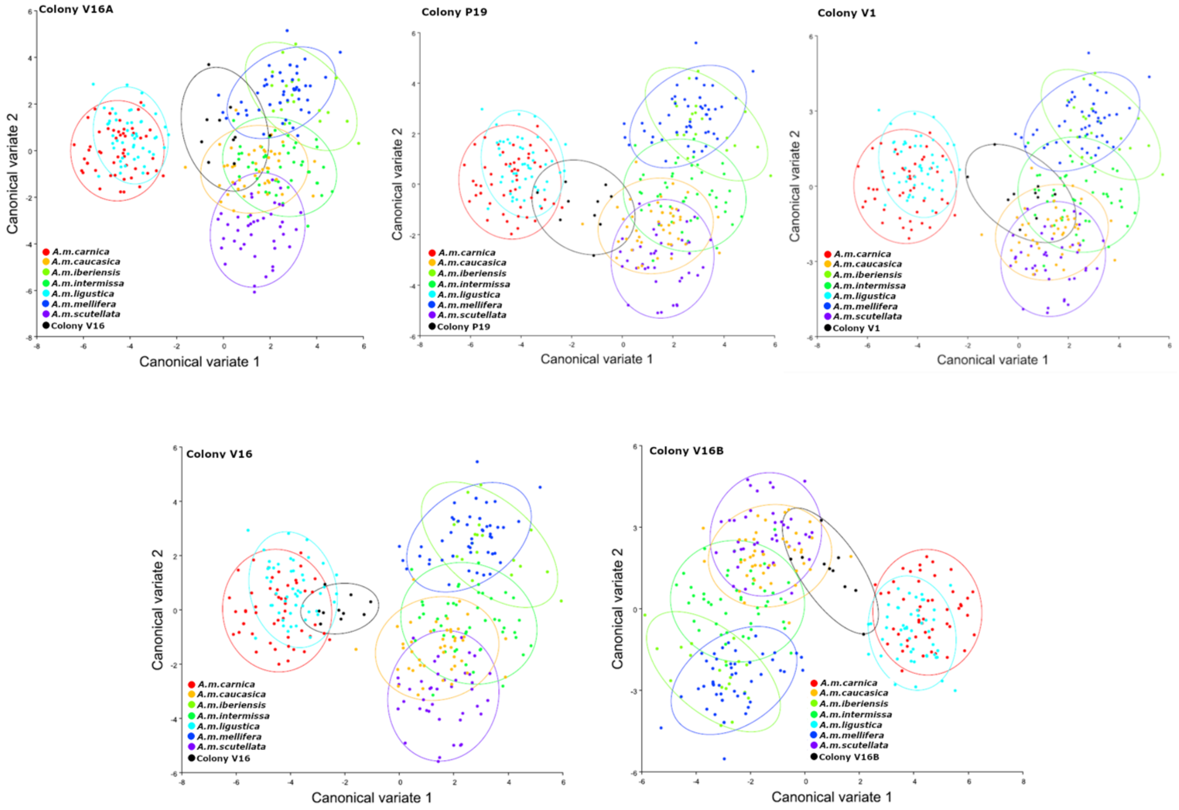

Figura suplementaria 1 - Análisis de variables canónicas. Las subespecies puras de *Apis mellifera* se representan en diferentes colores (rojo, naranja, verde, turquesa, azul claro, azul y violeta). La colonia analizada aparece en negro.
