# Supplementary material for: Morphometric and genetic characterization as tools for selection of Apis mellifera (Hymenoptera: Apidae) stocks in an area of natural hybridization in Argentina
Source: Front Insect Sci. 2023 Jan 17;2:1073999. doi: 10.3389/finsc.2022.1073999 (PMC10926486; doi:10.3389/finsc.2022.1073999)
Supplement: Supplementary file 3 [file Table_1.docx]

**Supplementary Table 1 - Mahalanobis Distances.** Genetic distance of the samples to each pure subspecies of *A. mellifera* (*A. m. carnica, A. m. caucasica, A. m. iberiensis, A. m. intermissa, A. m. ligustica, A. m. mellifera* and *A. m. scutellata*). * indicates the smallest genetic distances.

| Bees Subespecies | 07/03/2020 Sampling | | | | | 15/12/2020 Sampling | | | | |
| --- | --- | --- | --- | --- | --- | --- | --- | --- | --- | --- |
|  | P18 | P19 | V16 | V151 | V152 | V16A | P19 | V1 | V16 | V16B |
| *A. m. carnica* | 7,052 | 6,186 | 7,092 | 5,470 | 7,403 | 6,484 | 4,946* | 6,747 | 5,413* | 6,267* |
| *A. m. caucasica* | 5,558* | 5,659* | 5,564* | 4,967* | 6,152* | 6,064* | 5,414* | 5,644* | 6,609 | 5,871* |
| *A. m. iberiensis* | 7,037 | 6,881 | 6,920 | 6,279 | 7,082 | 6,676 | 6,926 | 6,341 | 7,592 | 7,234 |
| *A. m intermissa* | 5,018* | 5,925* | 6,027* | 4,461* | 6,177* | 6,029* | 5,546* | 6,065* | 6,725 | 6,420* |
| *A. m. ligustica* | 6,955 | 5,979* | 6,895 | 5,476 | 6,976 | 6,691 | 5,179* | 6,431 | 5,973* | 6,456* |
| *A. m. mellifera* | 5,896* | 5,918* | 6,519* | 5,300 | 7,158 | 5,780* | 6,174 | 6,353 | 6,851 | 6,775 |
| *A. m. scutellata* | 5,321* | 6,322 | 7,109 | 4,596* | 6,230* | 6,824 | 6,063 | 6,382 | 7,496 | 6,816 |
